# 4,6-Diaryl-5,5-difluoro-1,3-dioxanes as chiral dopants for liquid crystal compositions

**DOI:** 10.3762/bjoc.20.246

**Published:** 2024-11-14

**Authors:** Maurice Médebielle, Peer Kirsch, Jérémy Merad, Carolina von Essen, Clemens Kühn, Andreas Ruhl

**Affiliations:** 1 Universite Claude Bernard Lyon 1, CNRS, INSA Lyon, CPE, ICBMS, UMR 5246, Bâtiment LEDERER, 1 rue Victor Grignard, 69100 Villeurbanne Cedex, France; 2 Merck Electronics KGaA, Frankfurter Str. 250, D-64293 Darmstadt, Germany; 3 Institute of Materials Science, Technical University of Darmstadt, Peter-Grünberg-Str. 2, D-64287 Darmstadt, Germany

**Keywords:** chiral dopant, chirality, cholesteric phase, diols, fluorine, helical twisting power, liquid crystal

## Abstract

Two racemic *anti*-4,6-diphenyl-5,5-difluoro-1,3-dioxanes were prepared and the corresponding enantiomers were evaluated as potential new chiral dopants for liquid-crystal compositions.

## Introduction

**Figure 1:**
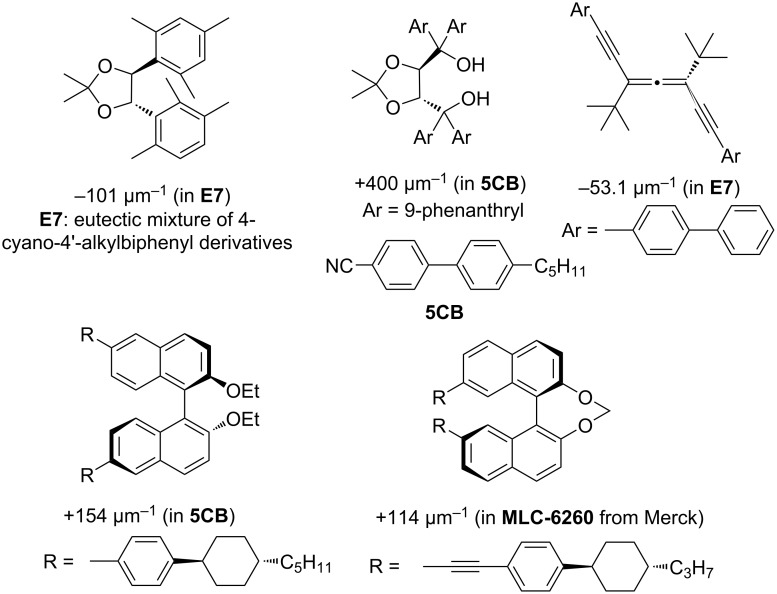
Selected examples of chiral dopants with high HTPs in their nematic host LC mixture.

Liquid crystals for use in liquid crystal displays (LCDs) have become one of the most prominent application areas of fluoroorganic chemistry [1–3]. In particular, cholesteric, i.e., chiral nematic, liquid crystals (LCs) are attractive for many display applications due to their chiroptical characteristics as well as the selective reflection of light giving rise to Bragg interference colors [4]. Cholesteric LCs can be obtained either from neat chiral mesogens, or through the addition of a chiral dopant to an achiral nematic liquid crystal [5–6]. The ability of the dopant to induce chirality in the nematic phase is defined as the *helical twisting power* [HTP; β = (pc)^−1^; with *p* the helical pitch and *c* the molar concentration]. The most common type of liquid crystal displays (LCD) is based on the so-called twisted nematic (TN) mode and requires only a quite small HTP (typically around 10–15 µm^−1^) with a low dopant concentration (around 0.1%) [1–2,7]. However, there are other display modes, such as super-twisted nematic (STN) LCDs which need a higher HTP or increased dopant concentrations [8–9]. TN and STN displays are still based on liquid crystals in the nematic or cholesteric mesophase. Even higher concentrations of chiral dopants with extremely high HTP tend to induce a Blue Phase, which is a cubic mesophase composed of double twist cylinders [10–11]. A prototype of a polymer-stabilized Blue Phase LCD with ultra-fast switching times has been presented in 2008 by Samsung [12]. Another class of materials including high HTP chiral dopants are cholesteric films prepared by the polymerization of reactive mesogens (RMs). They are used, e.g., for improving the viewing angle-dependency of the image quality of LCD panels or as polarizing reflectors [13–14]. For such applications, new dopants with very high HTP are in constant demand. There have been many reported chiral dopants with relatively high HTPs, including 4,5-diaryl-1,3-dioxolanes [15], TADDOLs [16–17], axially chiral alleno-acetylenes [18] and strained axially chiral 1,1’-binaphthyl derivatives [19–22] to cite a few (Figure 1 for selected examples).

As a general observation, the more elongated the liquid crystal-like shape of a molecule appears, the more efficient is the induction of chirality in a nematic host. Additionally, the location of the chiral substructure within the dopant molecule seems to play a role. A more ‘central’ location within in the mesogenic core structure corresponds to a higher HTP of the resulting chiral compound. Molecular structures like those depicted in Figure 1 suggest that low conformational flexibility in combination with π-stacking interactions between the aryl moieties of the dopant and those of the LC mixture might be the key parameters for a high HTP. Significant efforts have been made exploring a wide range of chiral, twisted molecules such as binaphthyls [23–25], biphenyls [26–27], TADDOLs [16–17] and 1,2-diphenylethane-1,2-diol [28] to reveal possible relationships between the molecular structure of chiral dopants and their HTP value. However, quantitative structure–property relationships still remain elusive and are not well understood [29].

Recently we have become interested in the preparation of racemic [30] *anti*- and highly enantioenriched 2,2-difluoro-1,3-diols [30–32] through an acylative double catalytic kinetic resolution (DoCKR) process [33]. While the 1,3-diol motif is found in some natural products [34] with some fluorinated analogues [35–36], this motif is rarely found in LCs [37–39].

**Scheme 1:**
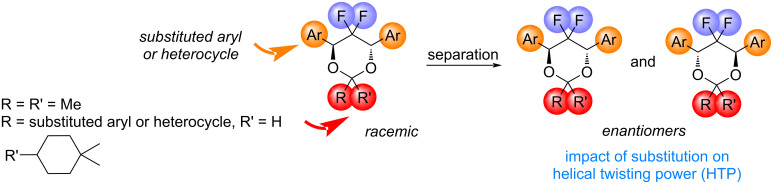
Structure–property relationship of 4,6-diheteroaryl-5,5-difluoro-1,3-dioxanes as potential chiral dopants.

Based on these observations and literature data, we embarked in the synthesis and evaluation of enantiomerically pure acetals derived from *anti*-2,2-difluoro-1,3-diols as potential chiral dopants. Our long-term goal would be to provide a structure–property relationship of this class of molecules by i) modification of the aryl and ii) and/or acetal moieties (Scheme 1) and to elucidate the possible role of fluorine on their physical properties, with comparison to their non-fluorinated analogs [38]. It is anticipated that the introduction of a CF_2_ could eventually stabilize the conformation of the six-membered ring via stereolectronic interactions of the CF σ* orbitals with the neighboring CH σ orbital [40]. The 180° FCCH dihedral would rigidify the six-membered ring.

**Scheme 2:**
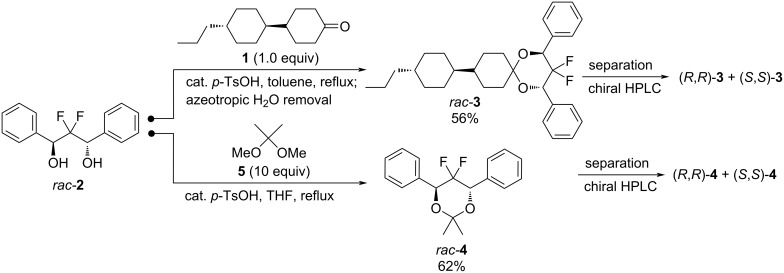
The syntheses of chiral 4,6-diphenyl-5,5-difluoro-1,3-dioxanes **3** and **4** as dopants for cholesteric liquid crystals.

We present here preliminary results with the diastereoselective synthesis of two 4,6-diphenyl-5,5-difluoro-1,3-dioxanes (*rac*-**3** and *rac*-**4**) and their separation into their corresponding enantiomers ((*R*,*R*)-**3** and (*S*,*S*)-**3**; (*R*,*R*)-**4** and (*S*,*S*)-**4**). *rac*-**3** and *rac*-**4** are obtained from readily available racemic 2,2-difluoro-1,3-diphenyl-1,3-propanediol (*rac*-**2**). These enantiomers were then evaluated as chiral dopants using two commercially available liquid crystal host mixtures (Host 1 and Host 2 from Merck KGaA) (Scheme 2).

## Results and Discussion

**Scheme 3:**
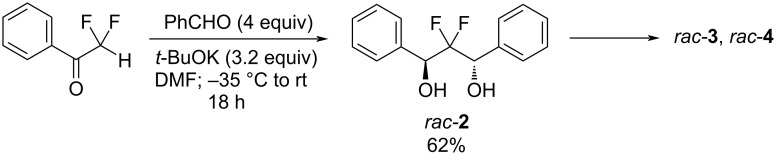
Synthesis of *rac*-**2** as precursor of *rac*-**3** and *rac*-**4**.

Racemic *anti*-2,2-difluoro-1,3-diol *rac*-**2** was easily prepared through a single step by an aldol-Tishchenko reaction [30] starting from commercially available difluoromethyl phenyl ketone [41–42] and an excess of benzaldehyde (4 equiv), under basic conditions (Scheme 3). Diol *rac-***2** was obtained in a 62% yield after recrystallization from CHCl_3_ with no need for chromatography purification. Ketalization of *rac*-**2** with liquid crystal-like ketone **1** [43] (in toluene) or 2,2-dimethoxypropane **5** (in THF) provided dioxanes *rac*-**3** and *rac*-**4** in 56% and 62% yields, respectively (Scheme 2).

**Figure 2:**
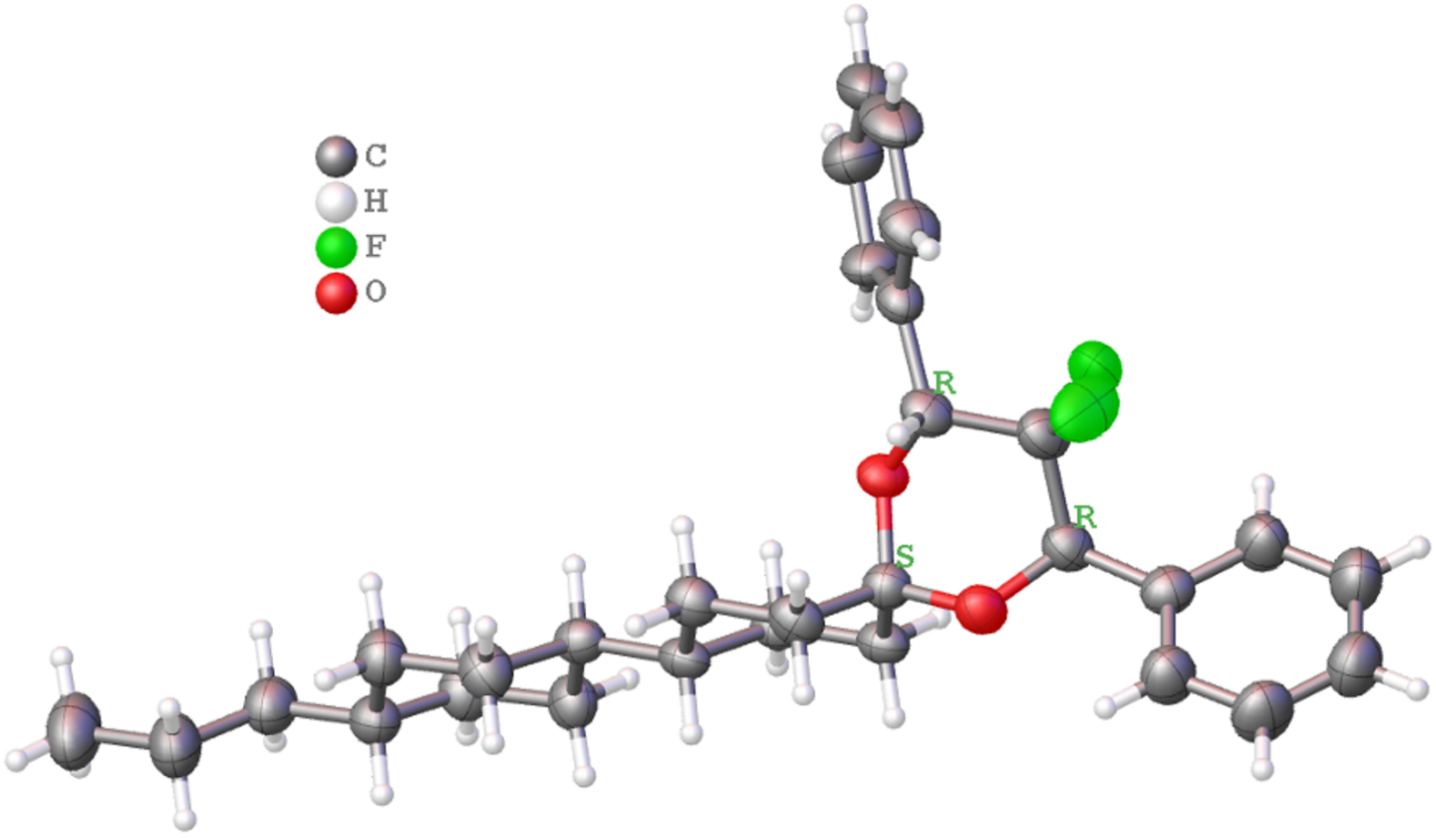
Configuration of (*R*,*R*)-**3** determined by X-ray crystallography.

**Figure 3:**
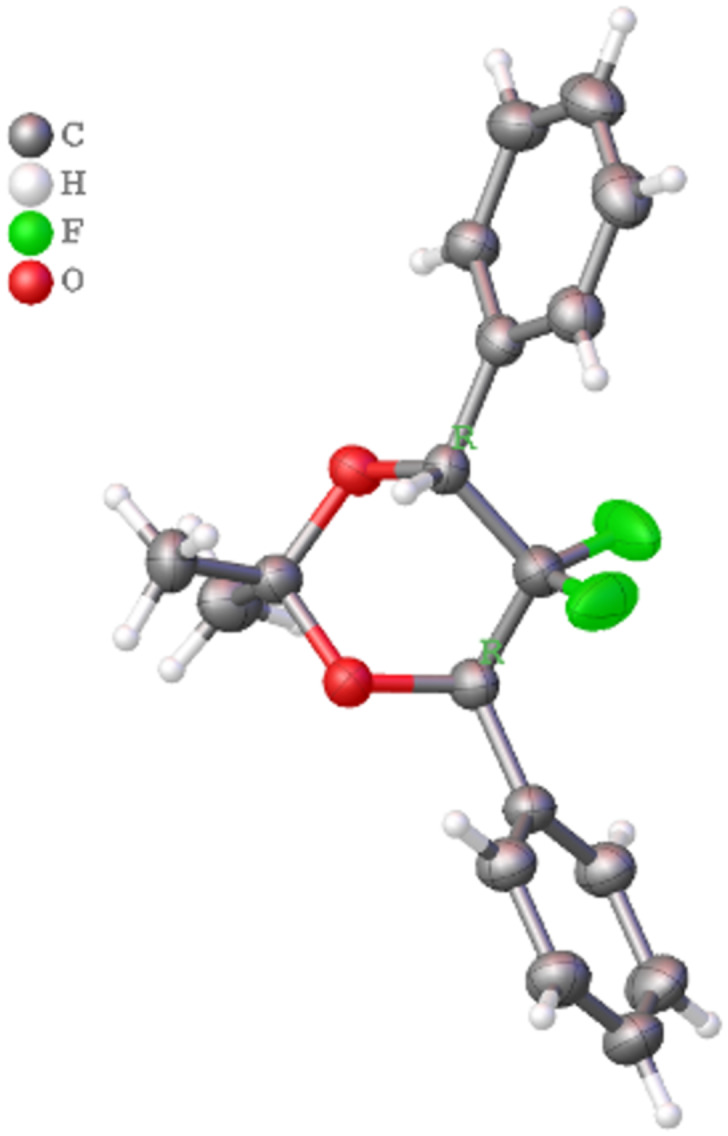
Configuration of (*R*,*R*)-**4** determined by X-ray crystallography.

Samples of *rac*-**3** and *rac*-**4** were separated by preparative HPLC on a chiral phase. Suitable crystals of all enantiomers (*R*,*R*)-**3**/(*S*,*S*)-**3**, (*R*,*R*)-**4**/(*S*,*S*)-**4** were obtained by slow evaporation from *n*-heptane solution and structures were determined by X-ray diffraction, which allowed the correlation of the chiroptical properties with the configuration (Figure 2 and Figure 3).

The HTP of all enantiomers were measured with two achiral nematic host mixtures (Host 1 and Host 2 from Merck) (Table 1). (*R*,*R*)-**3** and (*S*,*S*)-**3** with a more liquid crystal-like shape have higher HTP [16 µm^−1^ (Host 1), 38 µm^−1^ (Host 2)] than the dimethylacetal analogues (*R*,*R*)-**4** and (*S*,*S*)-**4** [8 µm^−1^ (Host 1), 15 µm^−1^ (Host 2)]. The difference between the two nematic host mixtures is their polarity: whereas Host 1 is only moderately polar (Δε = 4.0), the mixture Host 2 was developed for Blue Mode LCD application and is extremely polar. As compared to chiral dopants depicted in Figure 1, HTPs are lower and closest analogues for comparison could be the 4,5-diaryl-1,3-dioxolanes [15,28]. However, in particular the HTPs obtained for **3** are encouraging, since the values are still equal or higher than those of many commercially used chiral dopants [7]. Both compounds **3** and **4** have good solubilities in LCs hosts, and since their syntheses require few steps and are easily amenable to structural variation (e.g., of the aromatic moieties), there is a clear path towards even higher HTPs values.

**Table 1:** Comparison of the physical properties of enantiopures (*R*,*R*)-**3**, (*S*,*S*)-**3**, (*R*,*R*)-**4** and (*S*,*S*)-**4**.

No	Phase sequence^a^	Δ*H*_CI_^b^	*T*_NI,virt_^a,c^	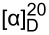 ^d^	HTP^e^

(*R*,*R*)-**3**	C 148 I	8500	−58.3	−5.20	−16 (Host 1) −38 (Host 2)
(*S*,*S*)-**3**	C 149 I	8500	−61.2	+ 6.80	+16 (Host 1) +38 (Host 2)
(*R*,*R*)-**4**	C 142 I	5100	–	−0.67	−8 (Host 1) −15 (Host 2)
(*S*,*S*)-**4**	C 142 I	5600	–	+ 0.60	+8 (Host 1) +15 (Host 2)

^a^The phase transition and virtual clearing temperatures *T*_NI,virt_ are given in °C; ^b^the melting enthalpies Δ*H*_CI_ are given in cal mol^−1^_; _^c^the values are extrapolated from a 5% w/w solution in ZLI-4792; ^d^the optical rotations are given in °; ^e^the helical twisting power (HTP) is given in µm^−1^; C = crystalline, I = isotropic. The "virtual" clearing temperatures *T*_NI_ were determined by linear extrapolation from a 5% w/w solution in the Merck mixture ZLI-4792 (*T*_NI_ = 92.8 °C, Δε = 5.3, Δ*n* = 0.0964). The extrapolated values are corrected empirically for differences in the order parameter. For the pure substances the phase transitions were identified by optical microscopy, and the corresponding temperatures by differential scanning calorimetry (DSC).

## Conclusion

In general, the *C*_2_-symmetric substructure in compounds (*R*,*R*)-/(*S*,*S*)-**3** and (*R*,*R*)-/(*S*,*S*)-**4** is quite efficient in inducing a chiral pitch into the nematic host mixtures Host 1 and Host 2. The extension of the chiral substructure (**4** →**3**) by the liquid crystal building block **1** into a more rod-like shape is further boosting the HTP by roughly a factor of 2. Moreover, the HTP of both compounds **3** and **4** depends strongly on the LC host mixture: for the more polar mixture Host 2 it is twice as high as for less polar Host 1.

## Supporting Information

File 1 Experimental procedures, analytical data and copies of NMR spectra.

## Data Availability

All data that supports the findings of this study is available in the published article and/or the supporting information of this article.
