# Supplementary material for: 4,6-Diaryl-5,5-difluoro-1,3-dioxanes as chiral dopants for liquid crystal compositions
Source: Beilstein J Org Chem. 2024 Nov 14;20:2940–5. doi: 10.3762/bjoc.20.246 (PMC11572010; doi:10.3762/bjoc.20.246)
Supplement: File 1 — Experimental procedures, analytical data and copies of NMR spectra. [file Beilstein_J_Org_Chem-20-2940-s001.pdf]

BEILSTEIN JOURNAL OF ORGANIC CHEMISTRY

Supporting Information

for

4,6-Diaryl-5,5-difluoro-1,3-dioxanes as chiral dopants for liquid crystal compositions

Maurice Médebielle, Peer Kirsch, Jérémy Merad, Carolina von Essen, Clemens Kühn and Andreas Ruhl

Beilstein J. Org. Chem. **2024,** *20,* 2940–2945. doi:10.3762/bjoc.20.246

Experimental procedures, analytical data and copies of NMR spectra

License and Terms: This is a supporting information file under the terms of the Creative Commons Attribution License (https://creativecommons.org/licenses/by/4.0). Please note that the reuse, redistribution and reproduction in particular requires that the author(s) and source are credited and that individual graphics may be subject to special legal provisions.
The license is subject to the *Beilstein Journal of Organic Chemistry* terms and conditions: (https://www.beilstein-journals.org/bjoc/terms)

Experimental – General methods S3

Experimental procedures S4

Synthesis of *rac*-**2** S4

Synthesis of *rac*-**3** S5

Deracemization of *rac*-**3** S6

(*R*,*R*)-**3** S7

(*S*,*S*)-**3** S8

Synthesis of *rac*-**4** S9

Deracemization of *rac*-**4** S10

(*S*,*S*)-**4** S10

(*R*,*R*)-**4** S11

NMR spectra S13

References S24

Experimental

General methods. All reactions were performed under an inert atmosphere of argon. Unless otherwise stated, all reagents were obtained from commercial suppliers and used without further purification. Experiments were run in dry solvents purchased from Sigma-Aldrich. ^{1}H, ^{19}F and ^{13}C NMR were recorded on Bruker Avance 300 or 400 spectrometers at 298 K (in $CDCl_3$ unless otherwise mentioned). Chemical shifts (δ) are expressed in parts per million (ppm) relative to residual peak of solvent (^{1}H NMR: 7.26 ppm for $CHCl_3$, ^{13}C NMR: 77.16 ppm for $CDCl_3$). Coupling constants (J) are given in Hertz. The following abbreviations and their combinations are used: s: singlet; d: doublet; t: triplet; m: multiplet; dd : doublet of doublets; td: triplet of doublets; dt: doublet of triplets; ddd: doublet of doublets of doublets. TLC were performed on Merck silica Gel 60 F254 plates with detection by UV light or $KMnO_4$ solution. Silica gel chromatography was performed on Macherey–Nagel silica gel 60M (0.04–0.063 mm) with solvents at technical grades. Mass spectra were recorded using a micrOTOF-Q [EI or ESI+, ESI−]. The analytical HPLC was conducted on RP-18e Purospher 250-4 1x 53; CH_3CN/MTBE 9:1, 60 mL·h^{-1}; sample concentration 1 mg/10 mL in eluent. The chiral analytical HPLC was conducted on Chiralpac AD-3R 150-4.6 1x Nr.37a; CH_3CN/H_2O 4:1, 60 mL h^{-1}; sample concentration 1 mg/10 mL in eluant. The liquid crystal host mixtures Host 1 (T_{NI} = 103.5°C, $\Delta\varepsilon$ = 4.0, Δn = 0.0880) and Host 2 (T_{NI} = 60°C) were obtained from Merck KGaA. The bicyclohexanone derivative **1** is a common liquid crystal building block and was obtained from Merck KGaA, Darmstadt.

Experimental procedures

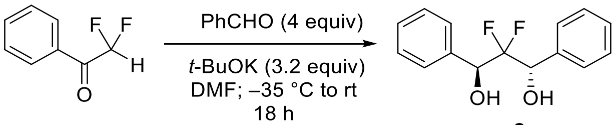

rac-**2**

Synthesis of *rac*-2 [1]: Benzaldehyde (2.72 g, 25.62 mmol) and difluoromethyl phenyl ketone (1.00 g, 6.40 mmol) were dissolved in anhydrous DMF (12.5 mL) under argon. The colorless solution was cooled down to –35 °C and a freshly prepared solution (12.5 mL) of *t*-BuOK in DMF (2.28 g, 20.32 mmol) was added dropwise. Solution turned yellow with formation of a fine white insoluble solid and after complete addition of the *t*-BuOK solution, the solution was maintained at –35 °C for 2 hours and warmed-up to room temperature and left overnight under argon atmosphere. The orange-red resulting solution was quenched with ice-water (60 mL) and extracted with diethyl ether (60 mL × 3), the combined extracts washed with a saturated NH_4Cl aqueous solution (60 mL), water (60 mL), dried over Na_2SO_4 and evaporated to dryness. The resulting yellow semi-solid solidified on standing and was recrystallized from $CHCl_3$ to furnish pure *rac*-**2**: 1.05 g (62%, 3.96 mmol). ^1H and ^{19}F NMR analysis of the crude (0.15 g) obtained after evaporation of the filtrate revealed the presence of *anti*- and *syn*-diols (*anti*/*syn*: 57.5/1.00).

^1H NMR (300 MHz, $CDCl_3$): δ = 7.38-7.46 (m, 10H), 5.08 (td, *J* = 11.3, 4.7 Hz, 2H), 2.97 (d, *J* = 4.9 Hz, 2H).

^{19}F NMR (282 MHz, $CDCl_3$): δ = -118.17 (t, *J* = 11.3 Hz, 2F).

HRMS (ESI-): calcd. for $C_{15}H_{13}F_2O_2$ $[M - H]^-$ 263.0889; found 263.0889

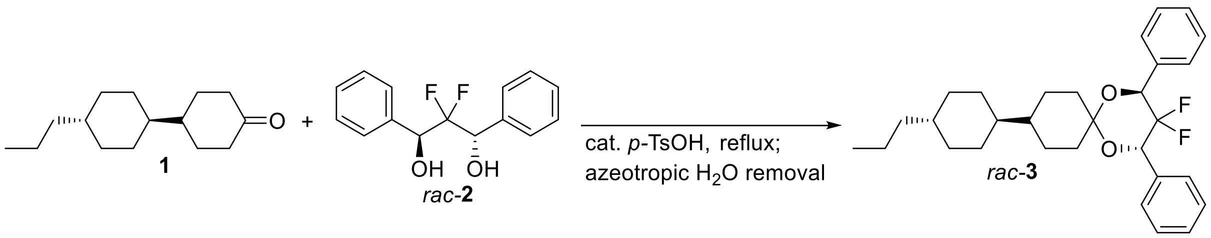

Synthesis of *rac*-3: A solution of **1** (910 mg, 4.09 mmol), *rac*-**2** (1.08 g, 4.08 mmol) and *p*-toluene sulfonic acid monohydrate (35 mg, 0.18 mmol) in toluene (50 mL) was refluxed under azeotropic water removal for 6 h. After cooling down, the mixture was washed with water (100 mL), dried over Na_2SO_4 and evaporated to dryness. The crude product was chromatographed over a short column (silica gel, toluene/*n*-heptane 1:1), furnishing 1.6 g of crude product. Further purification was achieved by crystallization from *n*-heptane: 1.12 g (56%, 100% purity by HPLC) of *rac*-**3**.

^{1}H NMR (400 MHz, $CDCl_3$): δ = 7.43-7.21 (m, 10H), 5.07 (ddd, *J* = 30.5, 17.3, 10.7 Hz, 2H), 2.27 (dt, *J* = 8.9, 3.1 Hz, 1H), 2.16-2.07 (m, 1H), 1.72-0.78 (m, 24H).

^{19}F NMR (377 MHz, $CDCl_3$): δ = -110.06 (dd, *J* = 17.9, 10.7 Hz), -110.69 (dd, *J* = 17.9, 10.6 Hz), -110.92 (dd, *J* = 17.2, 10.7 Hz), -111.55 (dd, *J* = 17.0, 10.7 Hz).

MS (EI): *m/z* (% rel. int.) = 41 (25), 55 (90), 69 (50), 107 (25), 140 (55), 141 (70), 152 (45), 210 (45), 229 (80), 230 (100), 301 (25), 397 (15), 468 (M^+, 20).

Deracemization of *rac*-3: *rac*-**3** (460 mg, 1.51 mmol) was recrystallized from *n*-heptane (3 mL) to furnish 250 mg of purified *rac*-**3** as colorless crystals (100% purity by HPLC). The racemate was separated by preparative chiral HPLC (Chiralpac AD-H; 12% MeOH in CH_3CN).

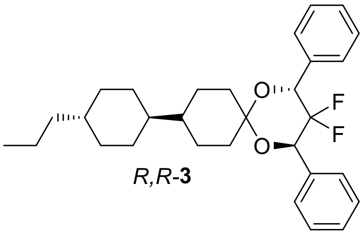

R,R-**3**

(*R,R*)-**3**: Mp. 149°C; virtual clearing temperature $T_{NI,virt}$ = −58.3°C (extrapolated from 5% w/w solution in ZLI-4792).

^{1}H NMR (400 MHz, $CDCl_3$): δ = 7.55-7.47 (m, 4H), 7.47-7.33 (m, 6H), 5.18 (ddd, *J* = 30.5, 17.3, 10.7 Hz, 2H), 2.39 (dt, *J* = 8.7, 3.3 Hz, 1H), 2.28-2.19 (m, 1H), 1.82-0.88 (m, 24H).

^{13}C NMR (101 MHz, $CDCl_3$): δ = 133.45, 133.22, 128.41, 128.24, 128.16, 127.19, 126.77, 102.98, 73.85, 73.27, 42.44, 42.29, 39.78, 37.55, 33.73, 33.51, 31.51, 30.36, 30.32, 26.35, 26.19, 20.02, 14.40.

^{19}F NMR (377 MHz, $CDCl_3$): δ = -110.06 (dd, *J* = 17.0, 10.7 Hz), -110.69 (dd, *J* = 17.4, 11.0 Hz), -110.92 (dd, *J* = 17.8, 10.7 Hz), -111.54 (dd, *J* = 18.0, 10.9 Hz).

EI-MS: *m/z* = 468.2865 ($M^{\cdot+}$), $C_{30}H_{38}F_2O_2$ requires 468.2840.

Optical rotation: $[\alpha]^{20}_{D}$= -5.20° (±3.4) (15 mg/10 mL CH_2Cl_2).

Helical twisting power (HTP): -16 µm^{-1} (Host 1), -38 µm^{-1} (Host 2).

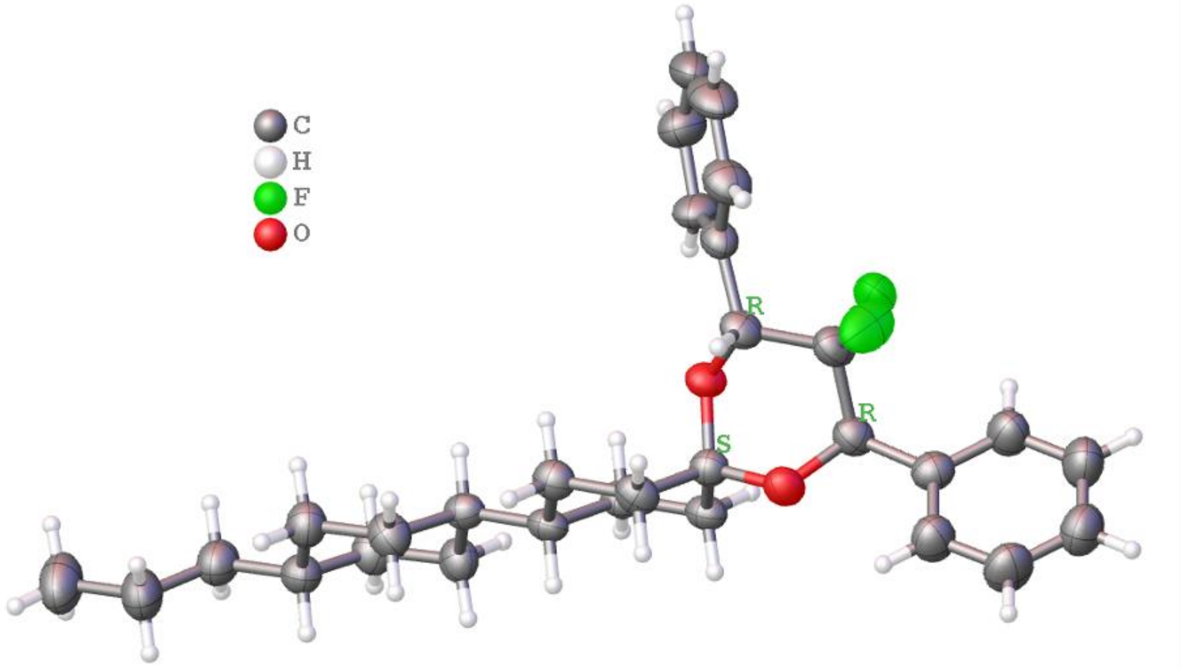

Figure 4: Crystal structure of (*R*,*R*)-**3** (thermal ellipsoids at 30% probability level).

Crystals of (*R*,*R*)-**3** ($C_{30}H_{38}F_2O_2$) were grown from *n*-heptane: crystal size (0.2411 × 0.0554 × 0.0361) mm^3, orthorhombic, $P2_12_12_1$, a = 5.7626(3) Å, b = 12.1105(5) Å, c = 37.3535(17) Å, $\alpha = \beta = \gamma = 90°$, V = 2606.8(2) $Å^3$, Z = 4, ρ_{calcd} = 1.194 g·cm^{-1}, R_{int} = 7.05%, R_1 = 4.27% for 2870 observed independent reflections (8.702° ≤ 2ϑ ≤ 103.42°), Hooft *y*: 0.16(12). Crystallographic data for the structure reported in this paper have been deposited with the Cambridge Crystallographic Data Centre as supplementary publication No. CCDC-2372810. Copies of the data can be obtained free of charge on application to CCDC, 12 Union Road, Cambridge CB2 1EZ, UK (fax: (+44) 1223-336-033; e-mail: deposit@ccdc.cam.ac.uk; http://www.ccdc.cam.ac.uk/).

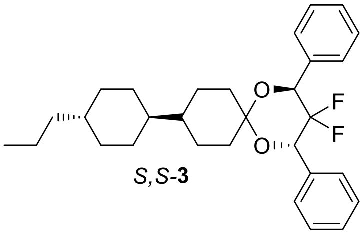

S,S-**3**

(*S,S*)-**3**: Mp. 148°C; virtual clearing temperature $T_{NI,virt}$ = −61.2°C (extrapolated from 5% w/w solution in ZLI-4792.

^{1}H NMR (400 MHz, $CDCl_3$): δ = 7.51-7.44 (m, 4H), 7.42-7.35 (m, 6H), 5.15 (ddd, *J* = 30.5, 17.3, 10.7 Hz, 2H), 2.38-2.3 (m, 1H), 2.23-2.19 (m, 1H), 1.77-0.83 (m, 24H).

^{13}C NMR (101 MHz, $CDCl_3$): δ = 133.45, 133.22, 128.41, 128.24, 128.16, 127.19, 126.77, 102.98, 73.85, 73.27, 42.43, 42.28, 39.78, 37.54, 33.72, 33.51, 31.50, 30.36, 30.31, 26.35, 26.18, 20.02, 14.39.

^{19}F NMR (377 MHz, $CDCl_3$): δ = -110.07 (dd, *J* = 17.8, 10.7 Hz), -110.69 (dd, *J* = 17.9, 10.7 Hz), -110.92 (dd, *J* = 17.1, 10.6 Hz), -111.55 (dd, *J* = 16.9, 10.7 Hz).

EI-MS: *m/z* = 468.2867 ($M^{\cdot+}$), $C_{30}H_{38}F_2O_2$ requires 468.2840.

Optical rotation: $[\alpha]_D^{20}$= +6.80° (±3.45) (15 mg/10 mL CH_2Cl_2).

Helical twisting power (HTP): +16 µm^{-1} (Host 1), +38 µm^{-1} (Host 2).

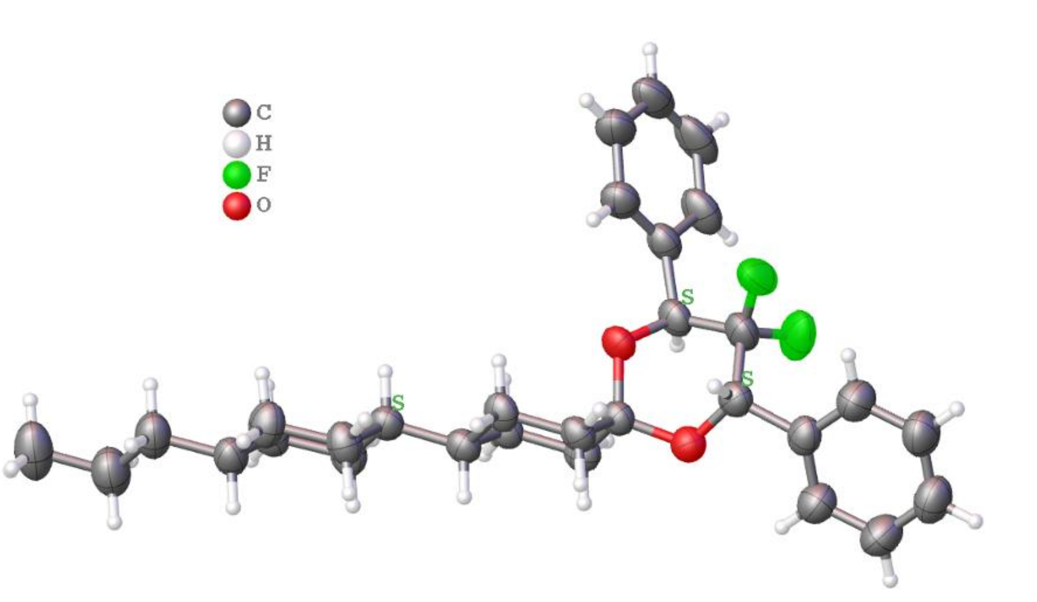

Figure 5: Crystal structure of (*S,S*)-**3** (thermal ellipsoids at 30% probability level).

Crystals of (*S,S*)-**3** ($C_{30}H_{38}F_2O_2$) were grown from *n*-heptane: crystal size (0.5931 × 0.1112 × 0.0691) mm^3, orthorhombic, $P2_12_12_1$, a = 5.7742(3) Å, b = 12.1276(5) Å, c = 37.3965(14) Å, $\alpha = \beta = \gamma = 90°$, V = 2618.78(19) Å^3, Z = 4, ρ_{calcd} = 1.189 g·cm^{-1}, R_{int} = 5.94%, R_1 = 6.76% for 5005 observed independent reflections (8.69° ≤ 2ϑ ≤ 147.91°), Hooft *y*: 0.0(2). Crystallographic data for the structure reported in this paper have been deposited with the Cambridge Crystallographic Data Centre as supplementary publication No. CCDC-2372813. Copies of the data can be obtained free of charge on application to CCDC, 12 Union Road, Cambridge CB2 1EZ, UK (fax: (+44) 1223-336-033; e-mail: deposit@ccdc.cam.ac.uk; http://www.ccdc.cam.ac.uk/).

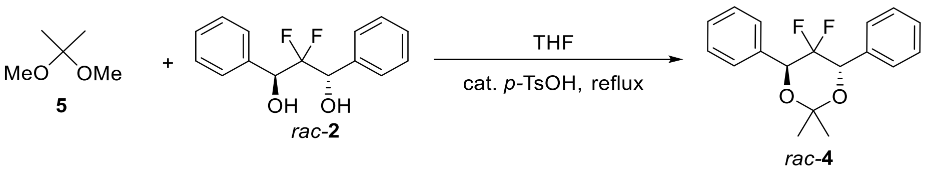

Synthesis of *rac*-4 [2]**:** A solution of **5** (3.96 g, 38 mmol), *rac*-**2** (1.0 g, 3.78 mmol) and *p*-toluene sulfonic acid monohydrate (84 mg, 0.44 mmol) in tetrahydrofuran (20 mL) was refluxed for 18 h. After cooling down, the mixture was poured into a saturated $NaHCO_3$ aqueous solution (50 ml), and extracted with diethyl ether (50 ml × 3). The combined extracts were dried over Na_2SO_4, filtered and evaporated to dryness. The crude product (1.2 g) was triturated with hot cyclohexane, insoluble solids filtered and filtrate concentrated, furnishing 0.71 g (2.34 mmol, 62%) of pure *rac*-**4**.

^{1}H NMR (300 MHz, $CDCl_3$): δ = 7.48-7.36 (m, 10H), 5.07 (t, J = 14.1 Hz, 2H), 1.60 (s, 6H).

^{19}F NMR (282 MHz, $CDCl_3$): δ = -110.96 (t, J = 13.8 Hz, 2F).

Deracemization of *rac*-4: *rac*-**4** (460 mg, 1.51 mmol) was recrystallized from *n*-heptane (3 mL) to furnish 250 mg of purified *rac*-**4** as colorless crystals (100% purity by HPLC). The racemate was separated by preparative chiral HPLC (Chiralpac AD-H 12% MeOH in CH_3CN).

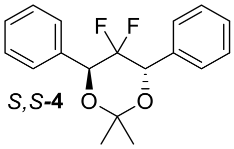

(*S,S*)-**4**: Mp. 142°C.

^{1}H NMR (500 MHz, $CDCl_3$): δ = 7.43-7.36 (m, 4H), 7.36-7.25 (m, 6H), 5.05 (t, *J* = 14.0 Hz, 2H), 1.53 (s, 6H).

^{19}F NMR (377 MHz, $CDCl_3$): δ = -110.91 (t, *J* = 13.8 Hz).

MS (EI): *m/z* (% rel. int.) = 43 (95), 51 (48), 77 (100), 89 (45), 105 (88), 107 (70), 114 (52), 120 (48), 140 (65), 141 (68), 198 (50), 229 (30), 246 (98), 304 (M^+, 10).

Optical rotation: $[\alpha]_D^{20}$= -0.60° (±1.67) (30 mg/10 mL CH_2Cl_2).

Helical twisting power (HTP): +8 µm^{-1} (Host 1), +15 µm^{-1} (Host 2).

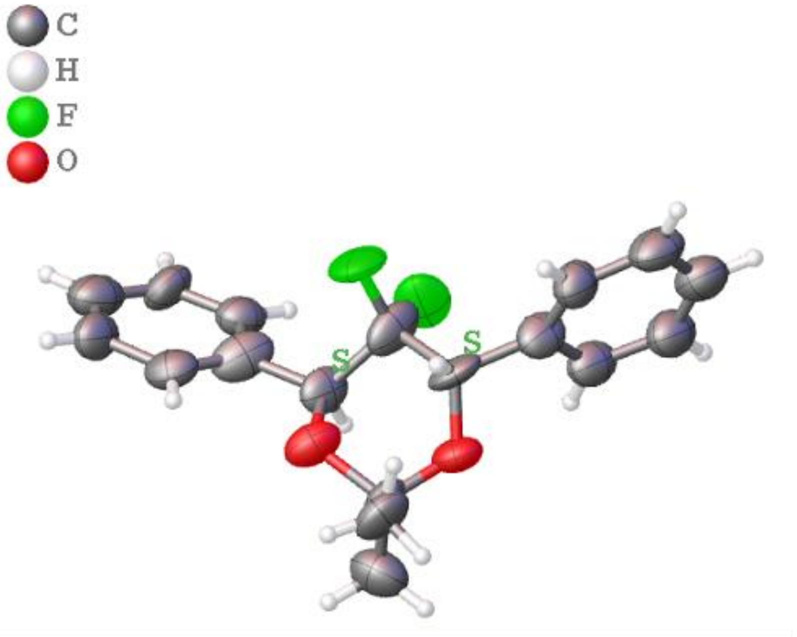

Figure 6: Crystal structure of (*S,S*)-**4** (thermal ellipsoids at 30% probability level).

Crystals of (*S,S*)-**4** ($C_{18}H_{18}F_2O_2$) were grown from *n*-heptane: crystal size (0.1023 × 0.0776 × 0.0066) mm^3, monoclinic, $P2_1$, a = 5.8928(10) Å, b = 7.8706(10) Å, c = 16.715(3) Å, α = 90.00°, β = 90.746(16)°, γ = 90.00°, V = 775.2(2) Å^3, Z = 2, ρ_{calcd} = 1.304 g·cm^{-1}, R_{int} = 7.4%, R_1 = 10.98% for 1856 observed independent reflections (10.58° ≤ 2ϑ ≤ 147.22°). The Friedel coverage is too low to reliably determine the absolute configuration. The correct assignment of the chiral centers can be confirmed by the correct assignment of the enantiomer and the overall consistency of the data. Crystallographic data for the structure reported in this paper have been deposited with the Cambridge Crystallographic Data Centre as supplementary publication No. CCDC-2372811. Copies of the data can be obtained free of charge on application to CCDC, 12 Union Road, Cambridge CB2 1EZ, UK (fax: (+44) 1223-336-033; e-mail: deposit@ccdc.cam.ac.uk; http://www.ccdc.cam.ac.uk/).

R,R-**4**

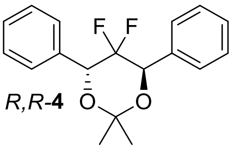

(*R,R*)-**4**: Mp. 142°C.

^{1}H NMR (500 MHz, $CDCl_3$): δ = 7.43-7.37 (m, 4H), 7.36-7.25 (m, 6H), 5.05 (t, J = 14.0 Hz, 2H), 1.53 (s, 6H).

^{19}F NMR (377 MHz, $CDCl_3$): δ = -110.92 (t, J = 13.8 Hz).

MS (EI): *m/z* (% rel. int.) = 43 (75), 51 (40), 77 (78), 89 (30), 105 (65), 107 (48), 114 (35), 120 (30), 140 (47), 141 (100), 198 (40), 229 (17), 246 (75), 304 (M$^+$, 5).

Optical rotation: $[\alpha]_D^{20}$= -0.67° (±1.67) (30 mg/10 mL CH_2Cl_2).

Helical twisting power (HTP): -8 µm^{-1} (Host 1), -15 µm^{-1} (Host 2).

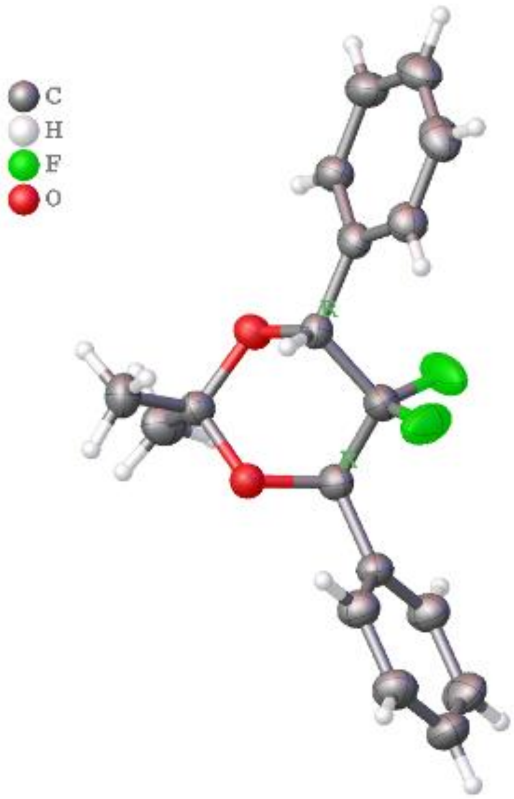

Figure 7: Crystal structure of (*R,R*)-**4** (thermal ellipsoids at 30% probability level).

Crystals of (*R,R*)-**4** ($C_{18}H_{18}F_2O_2$) were grown from *n*-heptane: crystal size (0.312 × 0.266 × 0.135) mm^3, monoclinic, $P2_1$, a = 5.9024(2) Å, b = 7.8749(3) Å, c = 16.7065(6) Å, α = 90.00°, β = 90.778(4)°, γ = 90.00°, V = 776.46(5) Å^3, Z = 2, ρ_{calcd} = 1.302 g·cm^{-1}, R_{int} = 2.65%, R_1 = 3.32% for 2358 observed independent reflections (10.59° ≤ 2ϑ ≤ 124.56°), Hooft *y*: 0.03(6). Crystallographic data for the structure reported in this paper have been deposited with the Cambridge Crystallographic Data Centre as supplementary publication No. CCDC-2372812. Copies of the data can be obtained free of charge on application to CCDC, 12 Union Road, Cambridge CB2 1EZ, UK (fax: (+44) 1223-336-033; e-mail: deposit@ccdc.cam.ac.uk; http://www.ccdc.cam.ac.uk/).

^{1}H NMR of *rac*-**2**

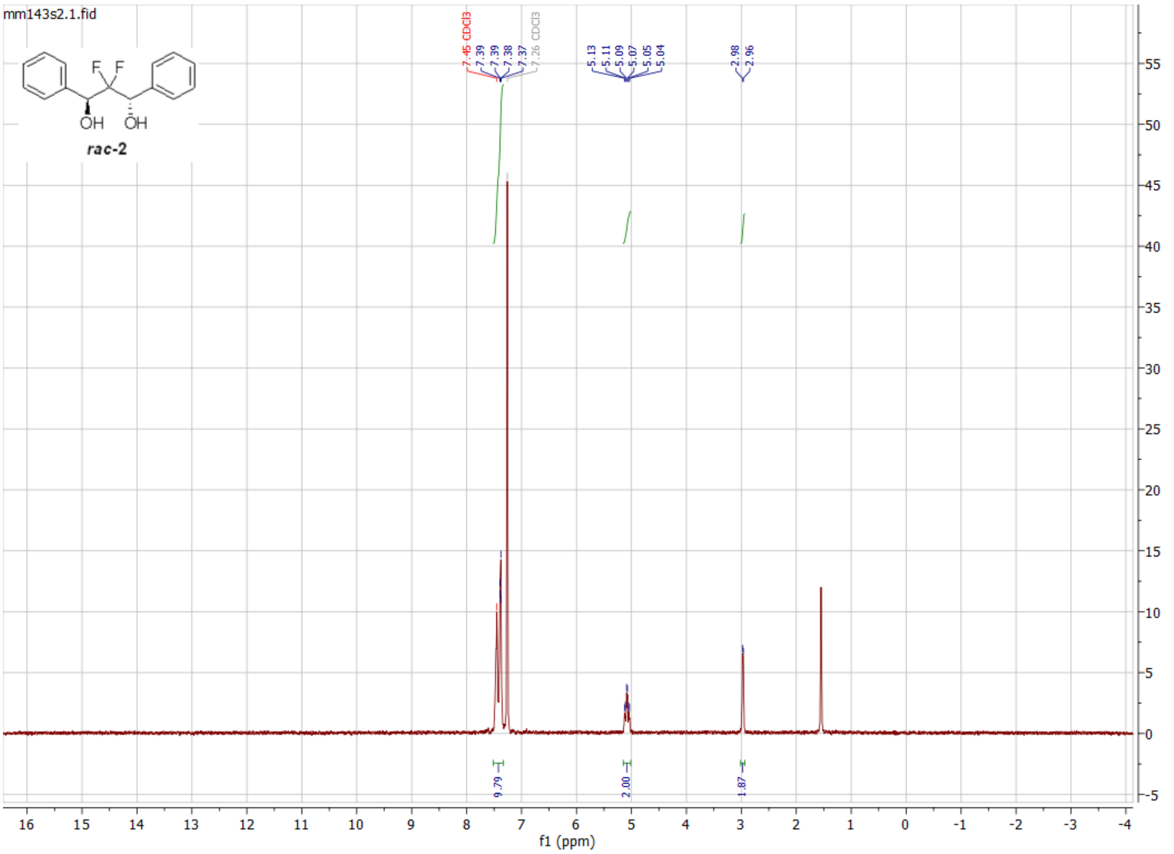

^{19}F NMR of *rac*-**2**

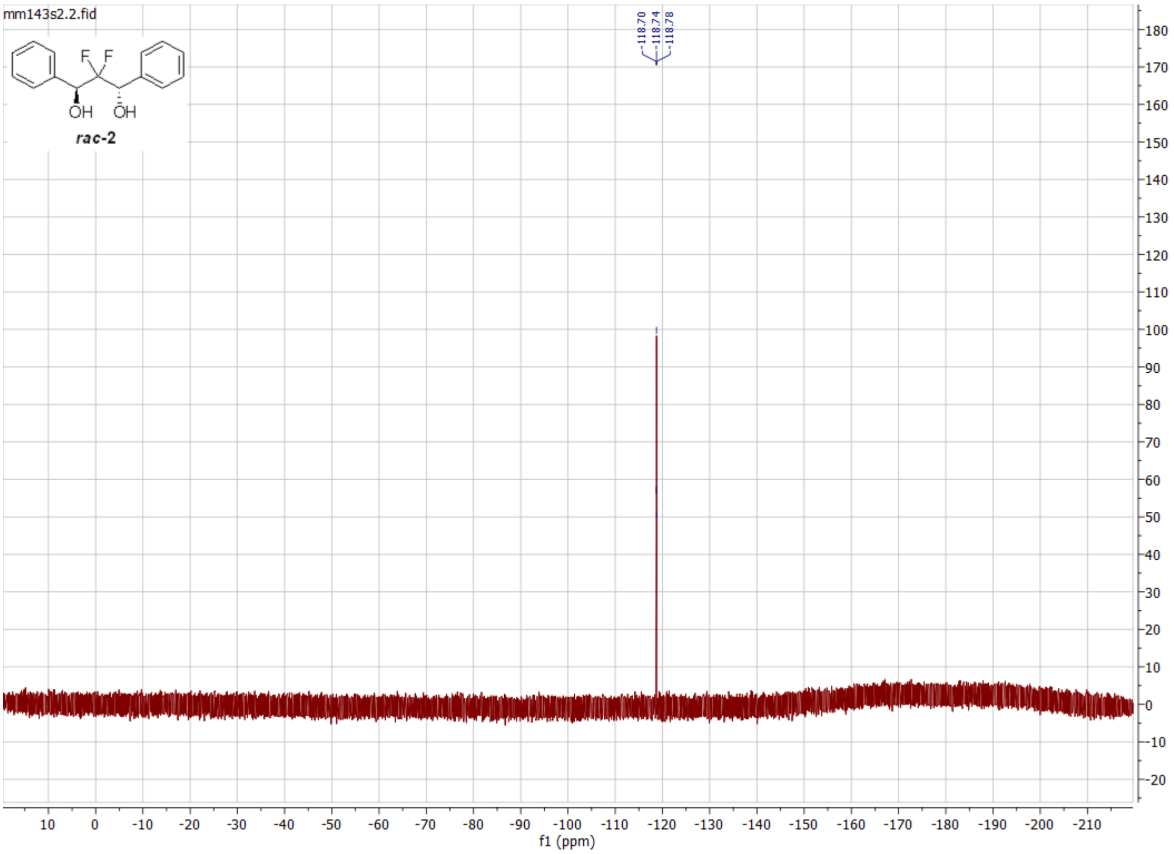

^{19}F NMR of *rac*-**2**

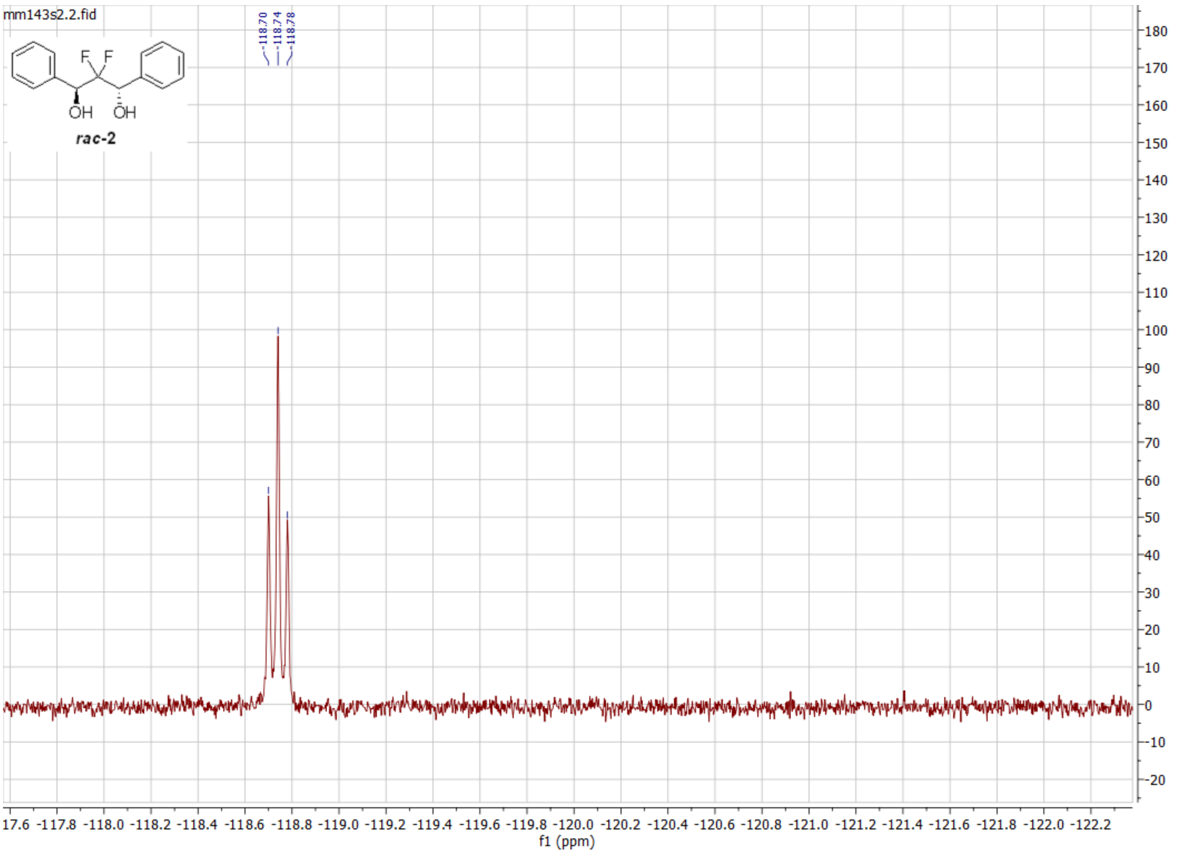

^{1}H NMR of *rac*-**3**

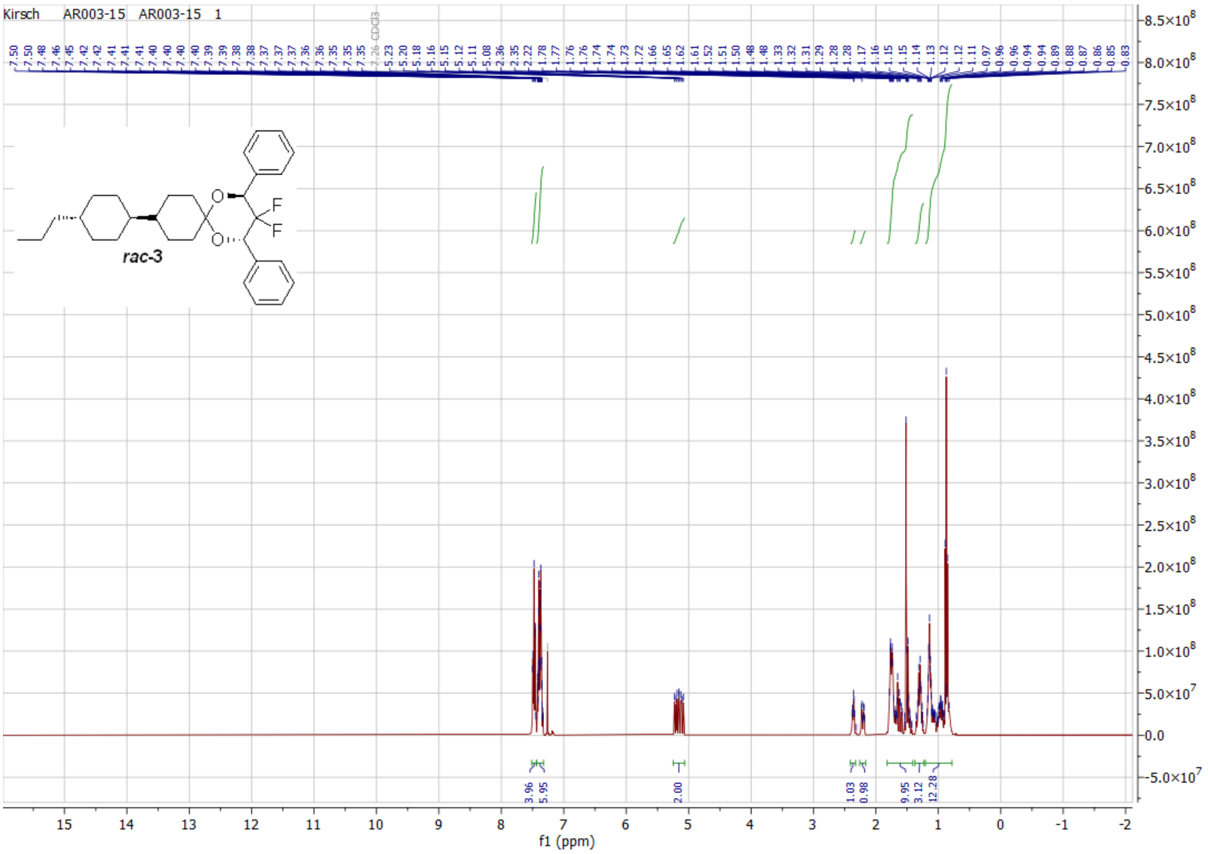

^{19}F NMR of *rac*-**3**

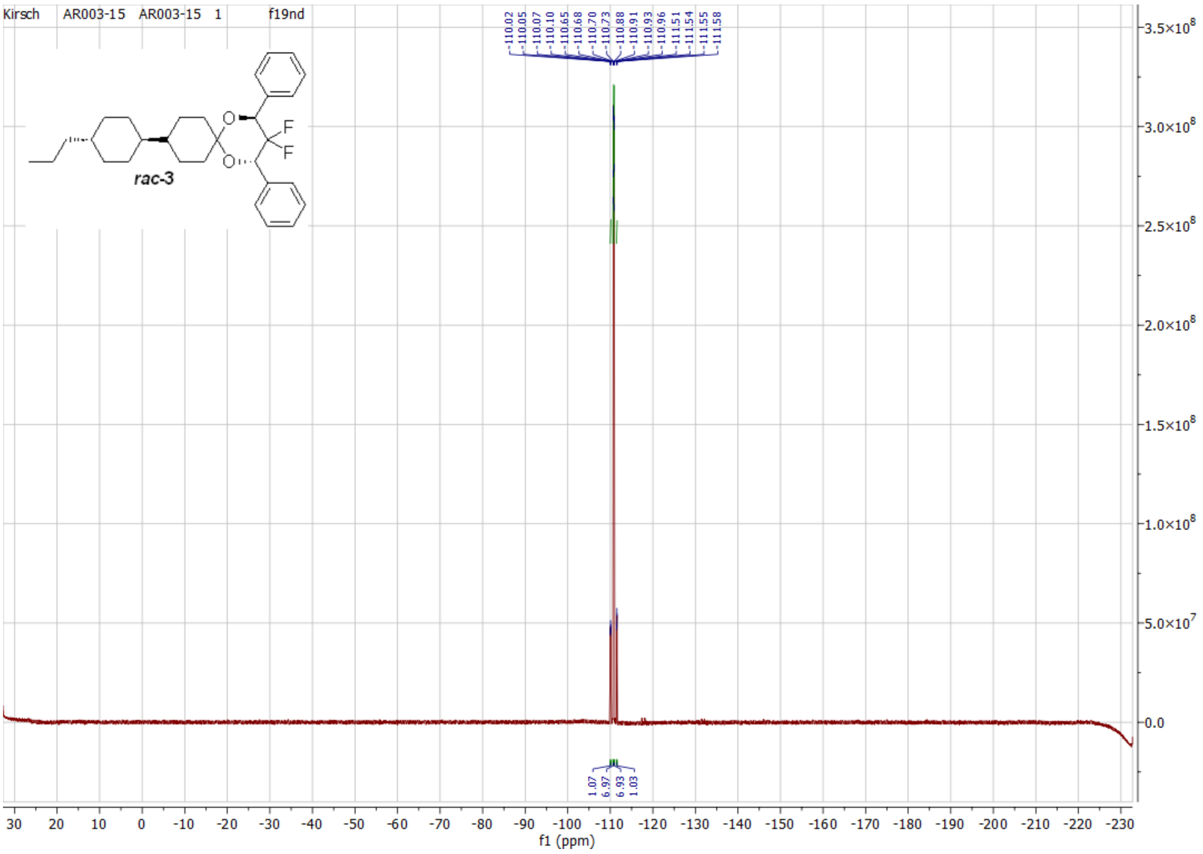

^{19}F NMR of *rac*-**3**

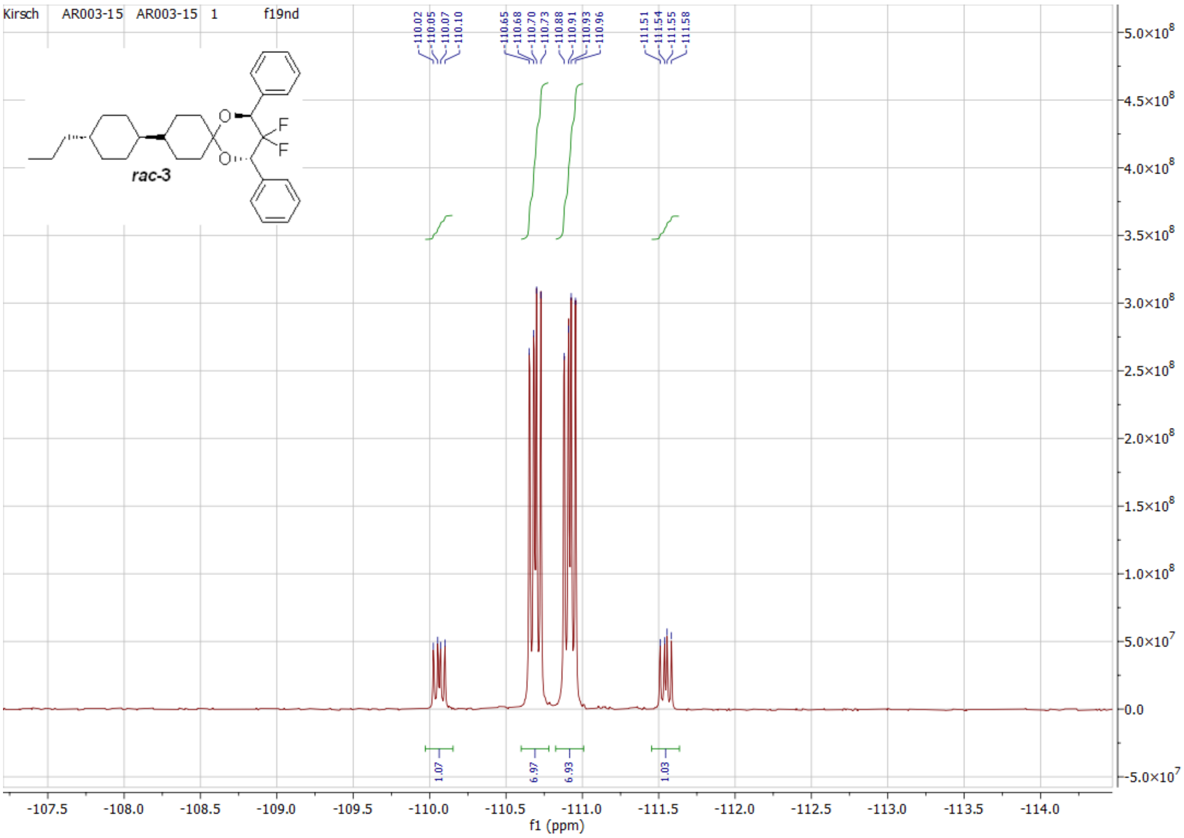

^{1}H NMR of (*R*,*R*)-**3**

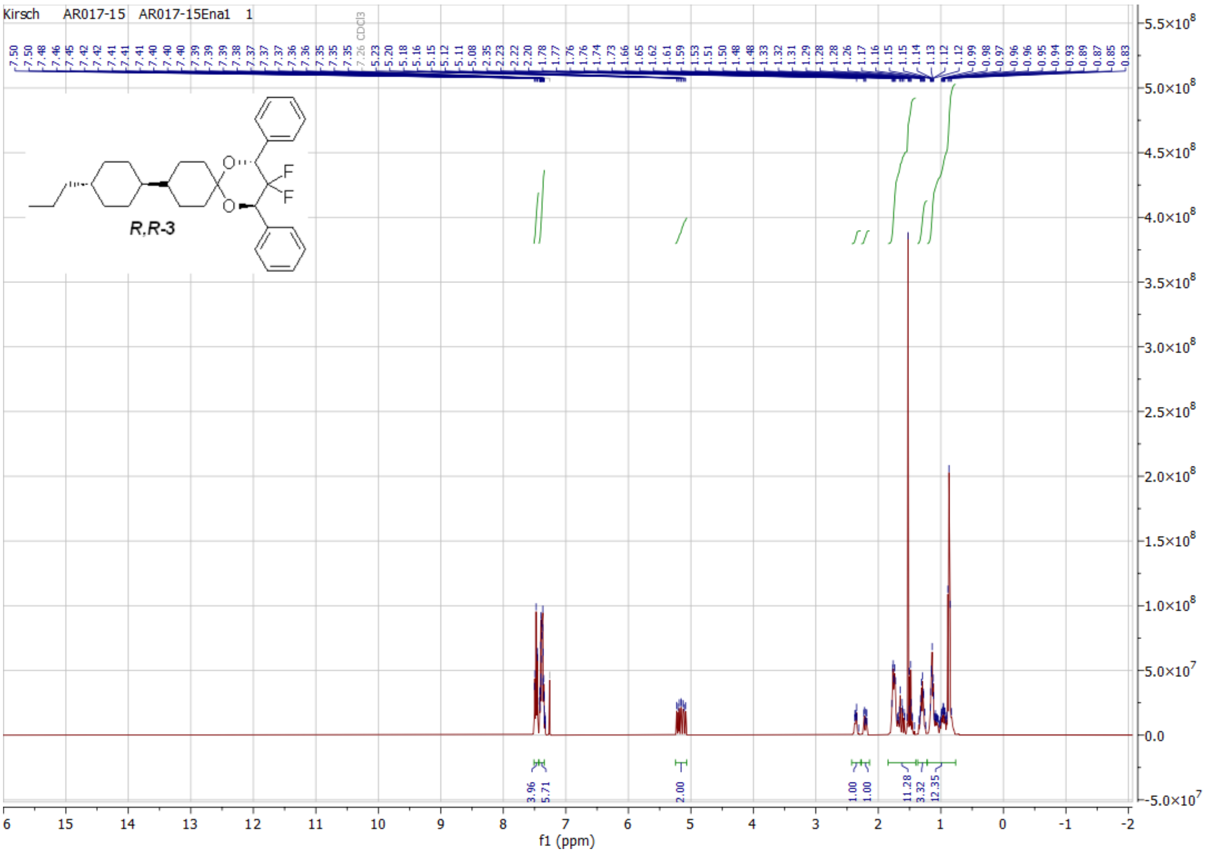

^{19}F NMR of (*R*,*R*)-**3**

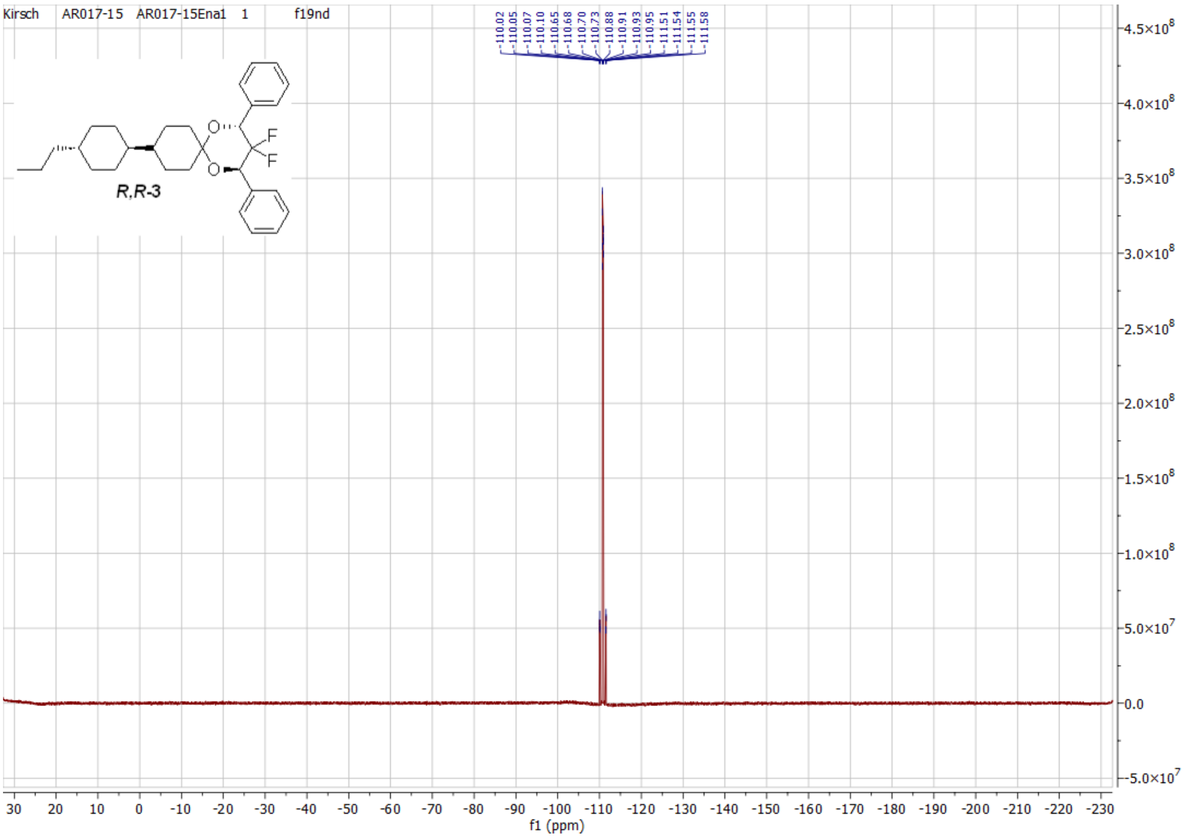

^{19}F NMR of (*R*,*R*)-**3**

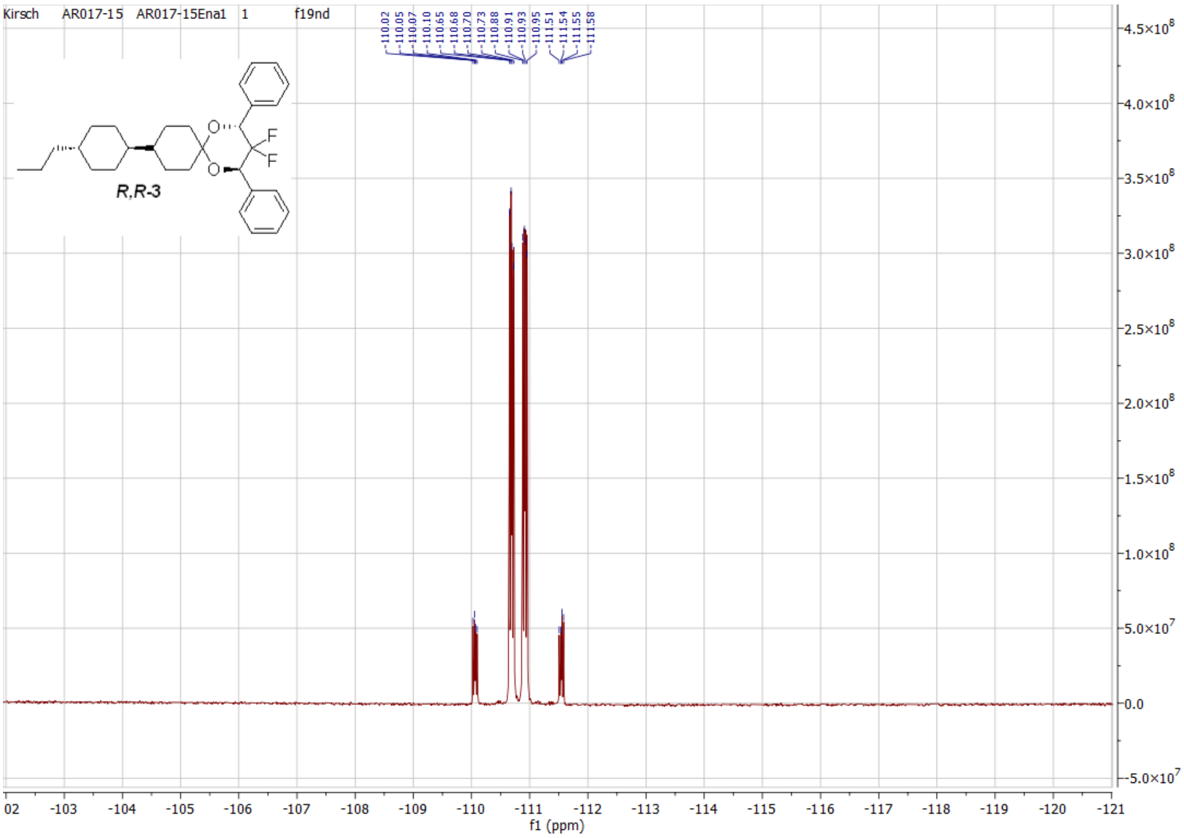

^{13}C NMR of (*R*,*R*)-**3**

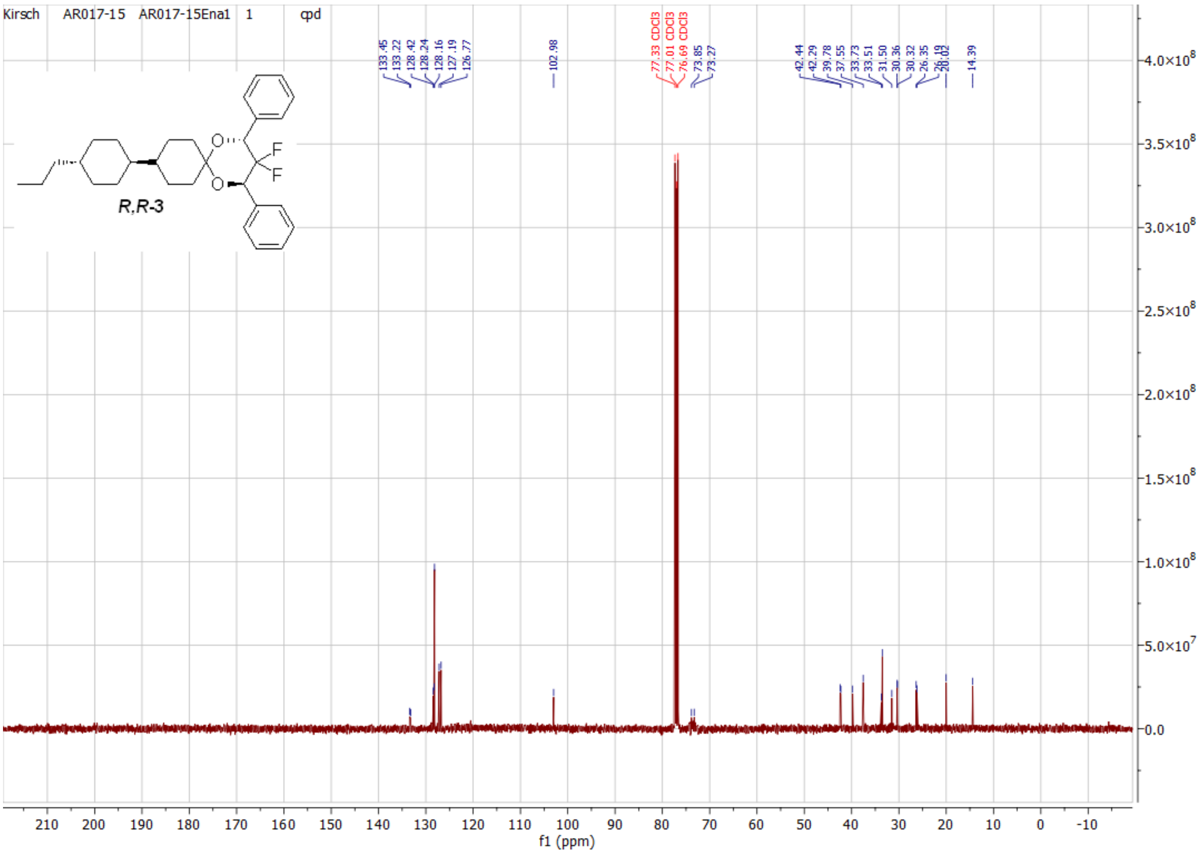

^{1}H NMR of (*S*,*S*)-**3**

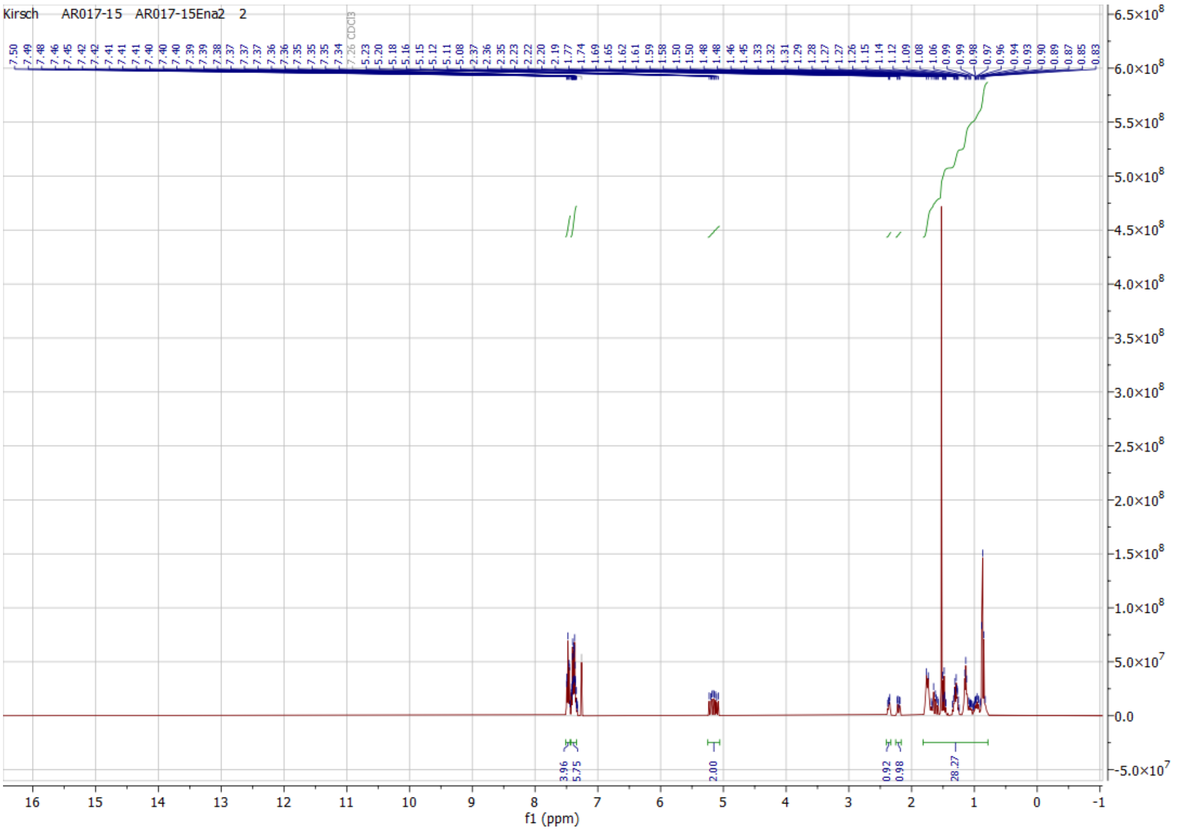

^{19}F NMR of (*S*,*S*)-**3**

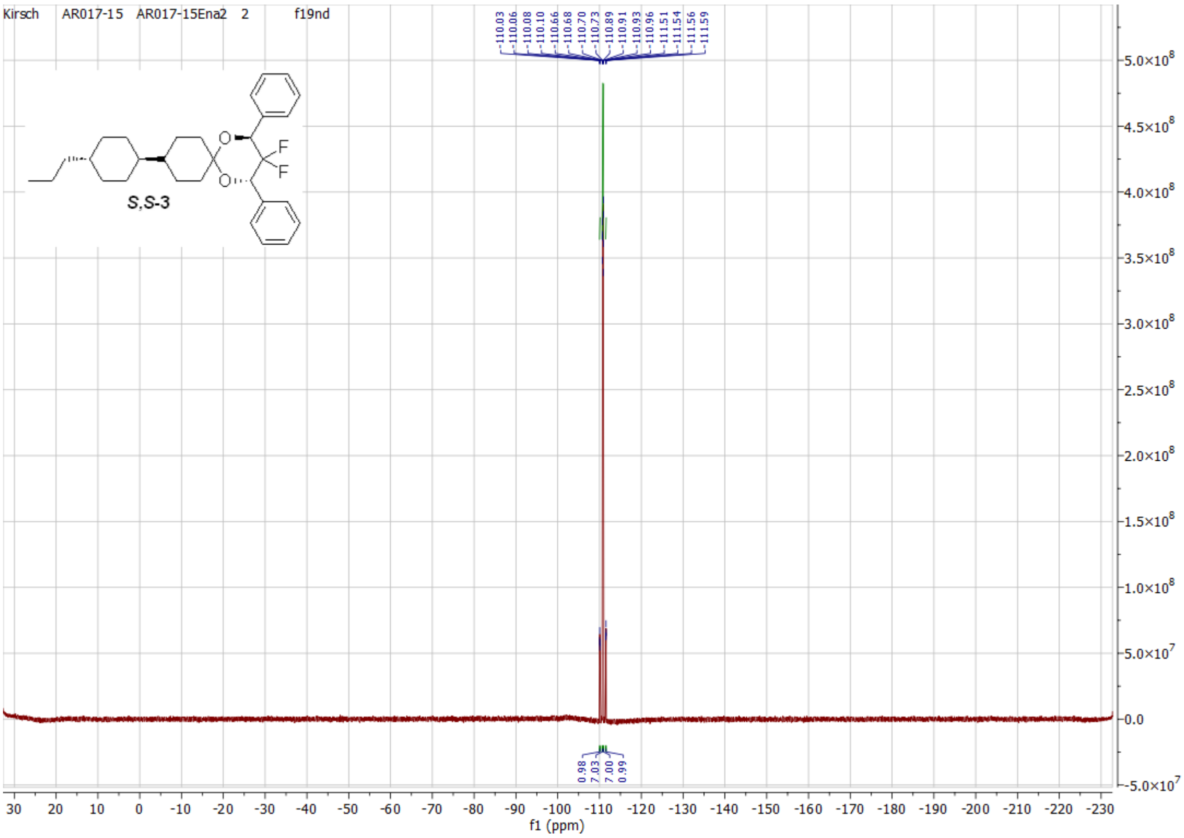

^{19}F NMR of (*S*,*S*)-**3**

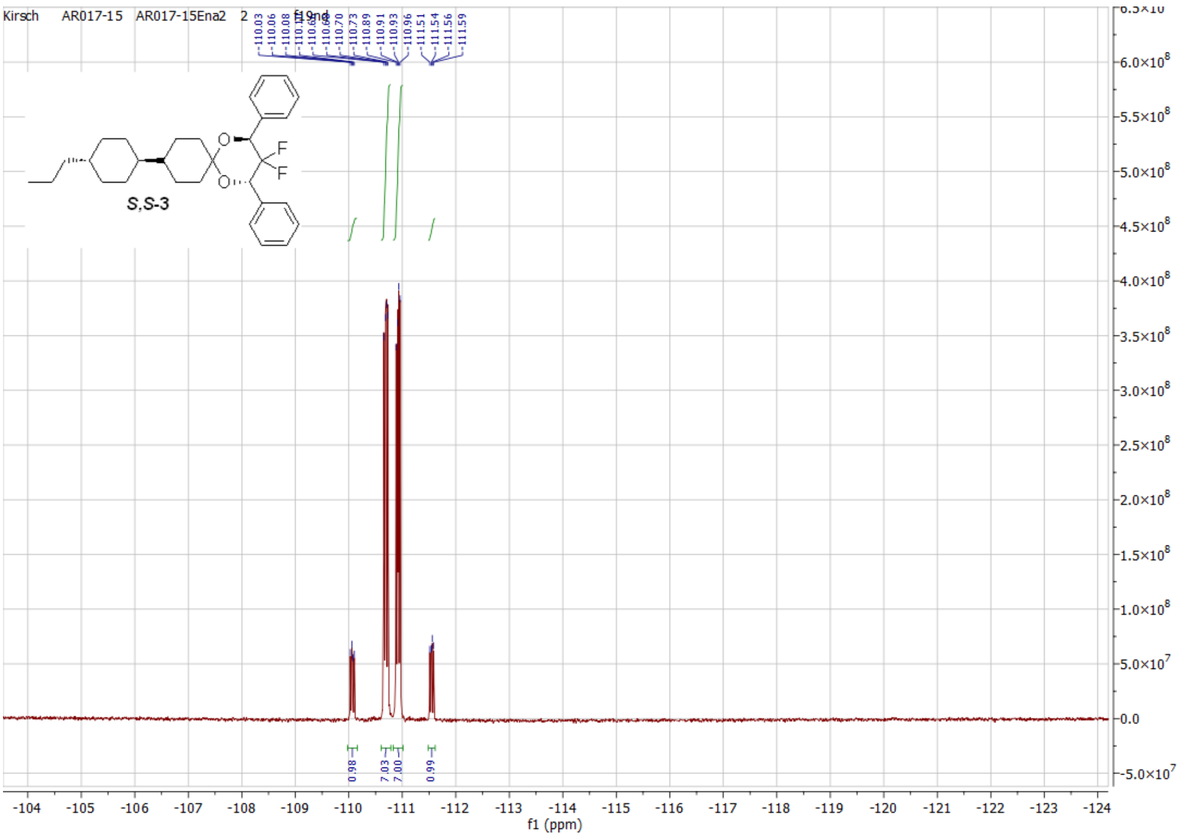

^{13}C NMR of (*S*,*S*)-**3**

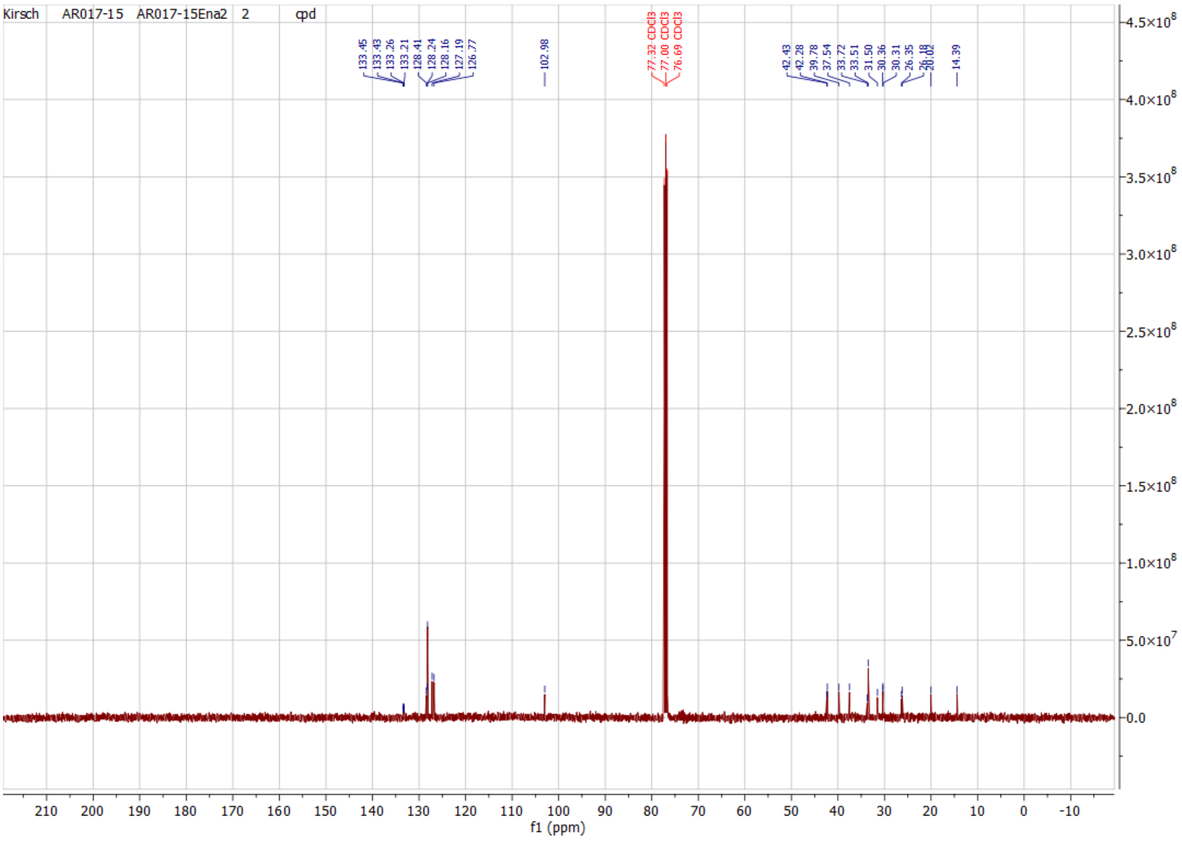

^{1}H NMR of *rac*-**4**

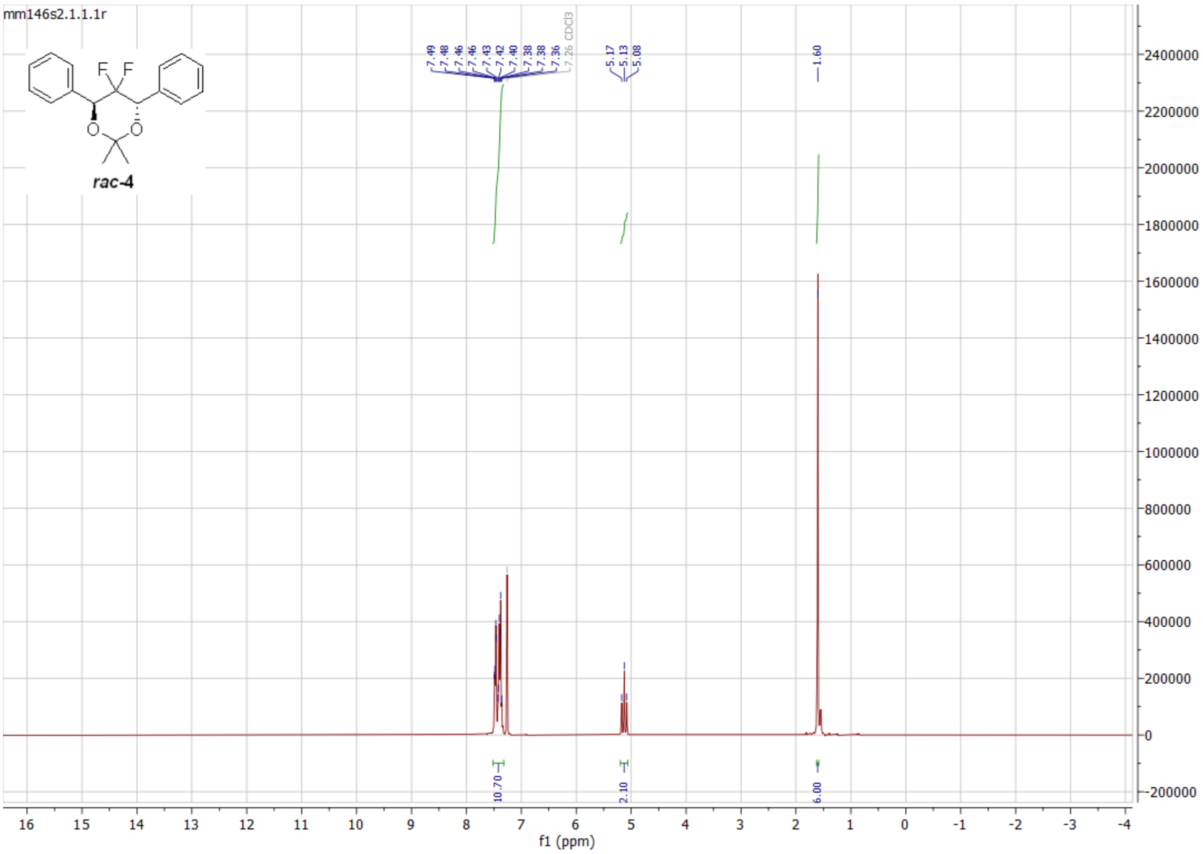

^{19}F NMR of *rac*-**4**

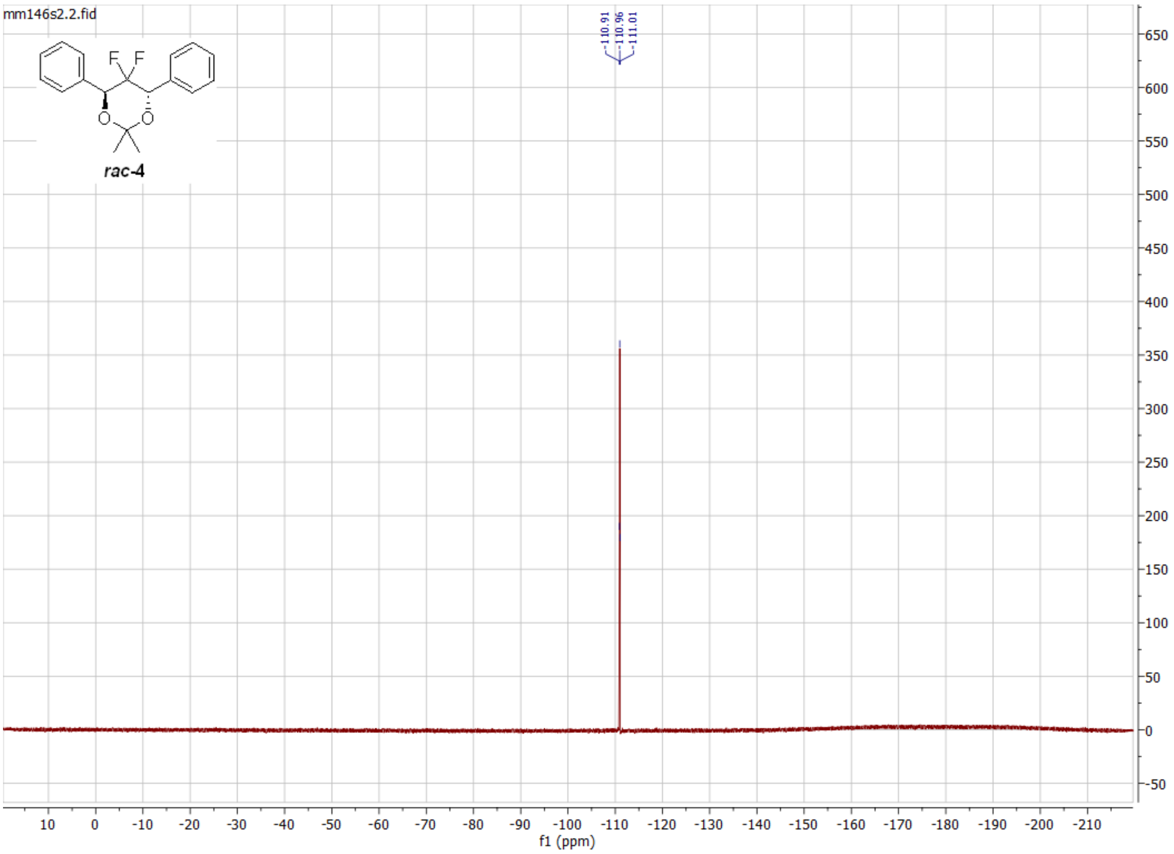

^{19}F NMR of *rac*-**4**

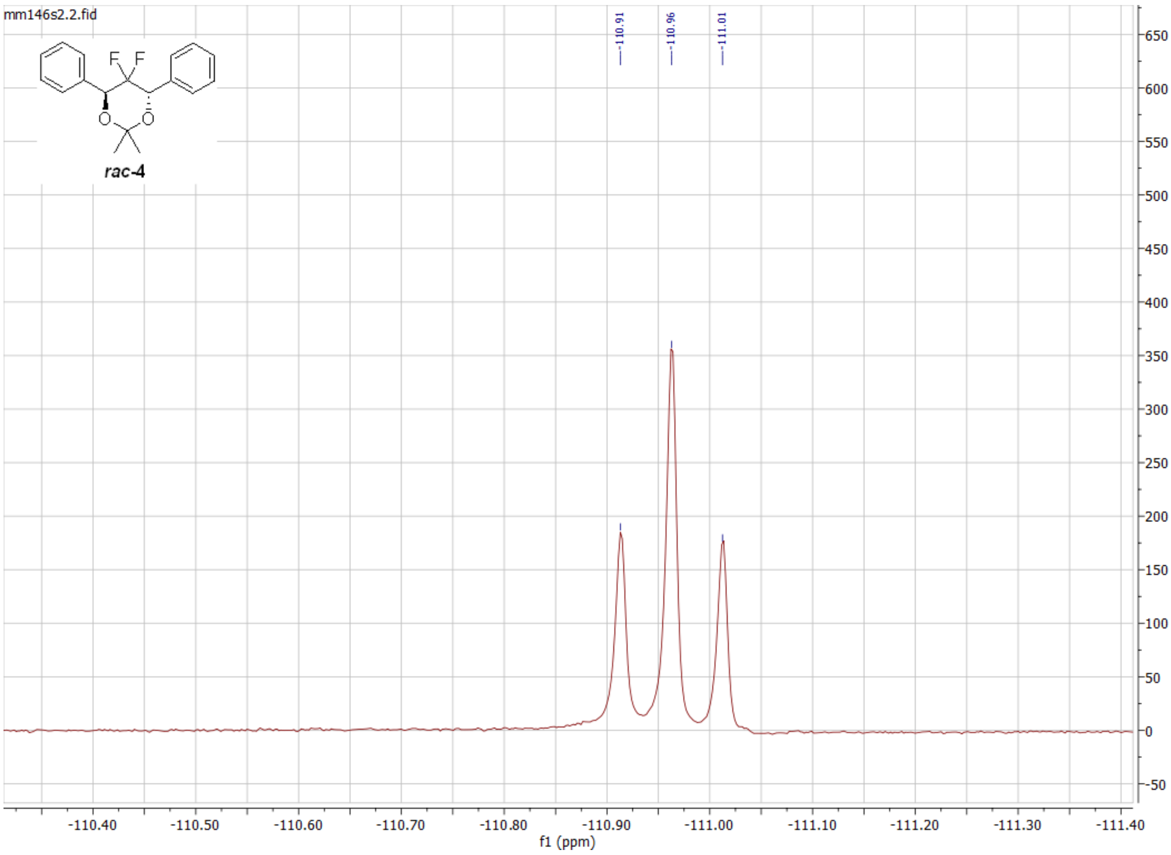

^{1}H NMR of (*S*,*S*)-**4**

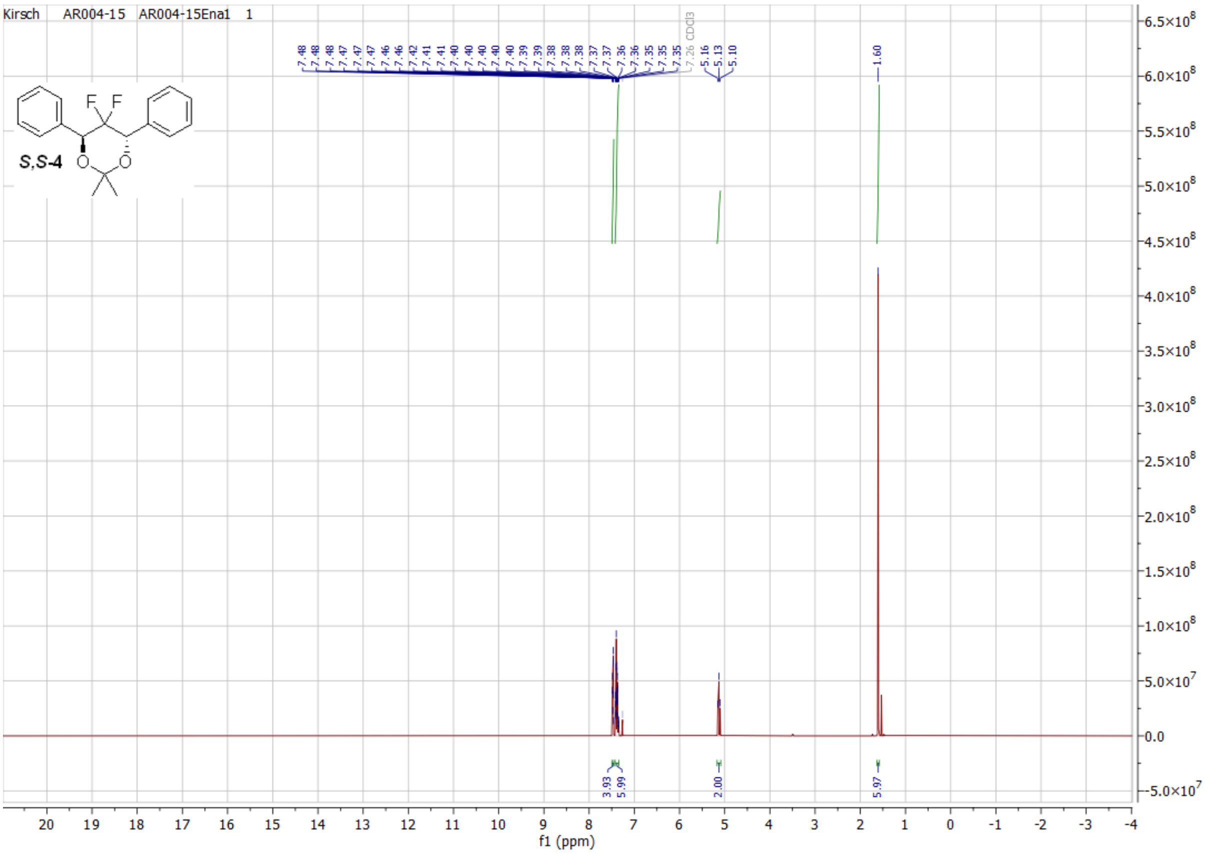

^{19}F NMR of (*S*,*S*)-**4**

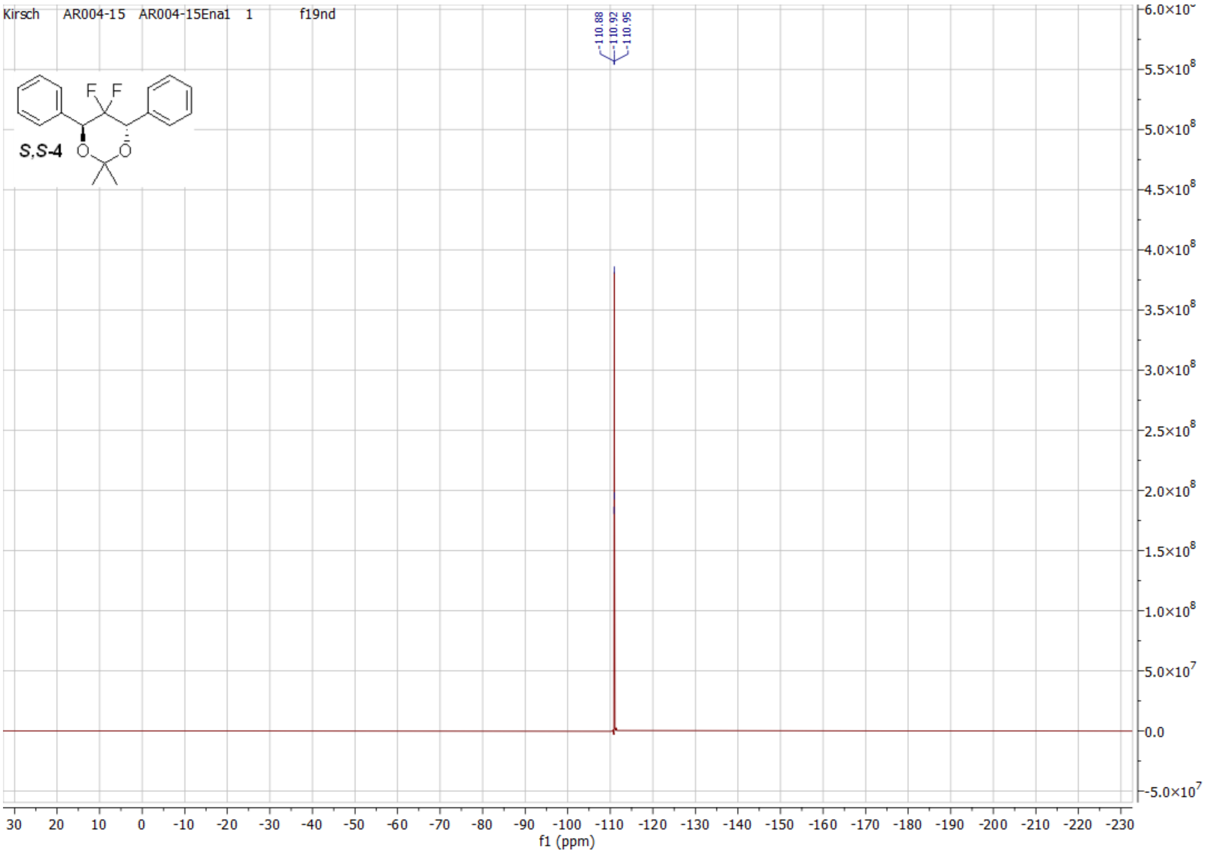

^{19}F NMR of (*S*,*S*)-**4**

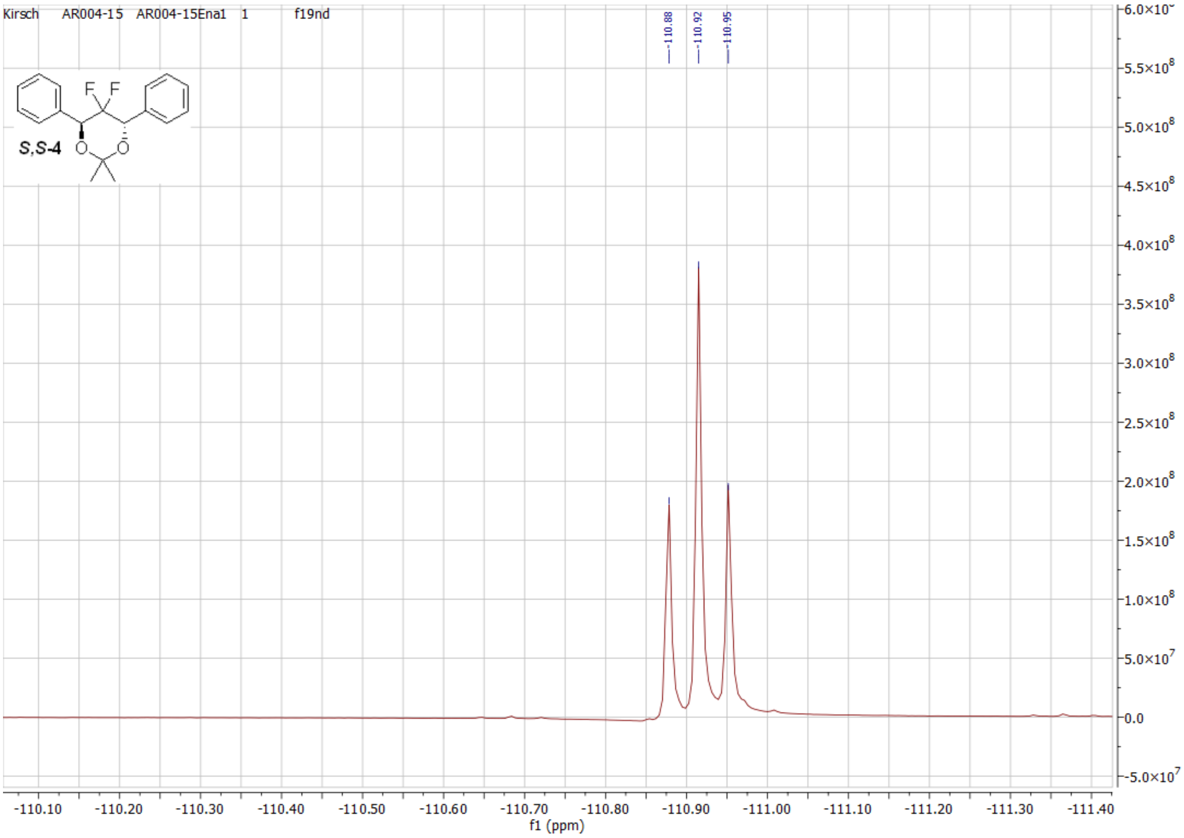

^{1}H NMR of (*R*,*R*)-**4**

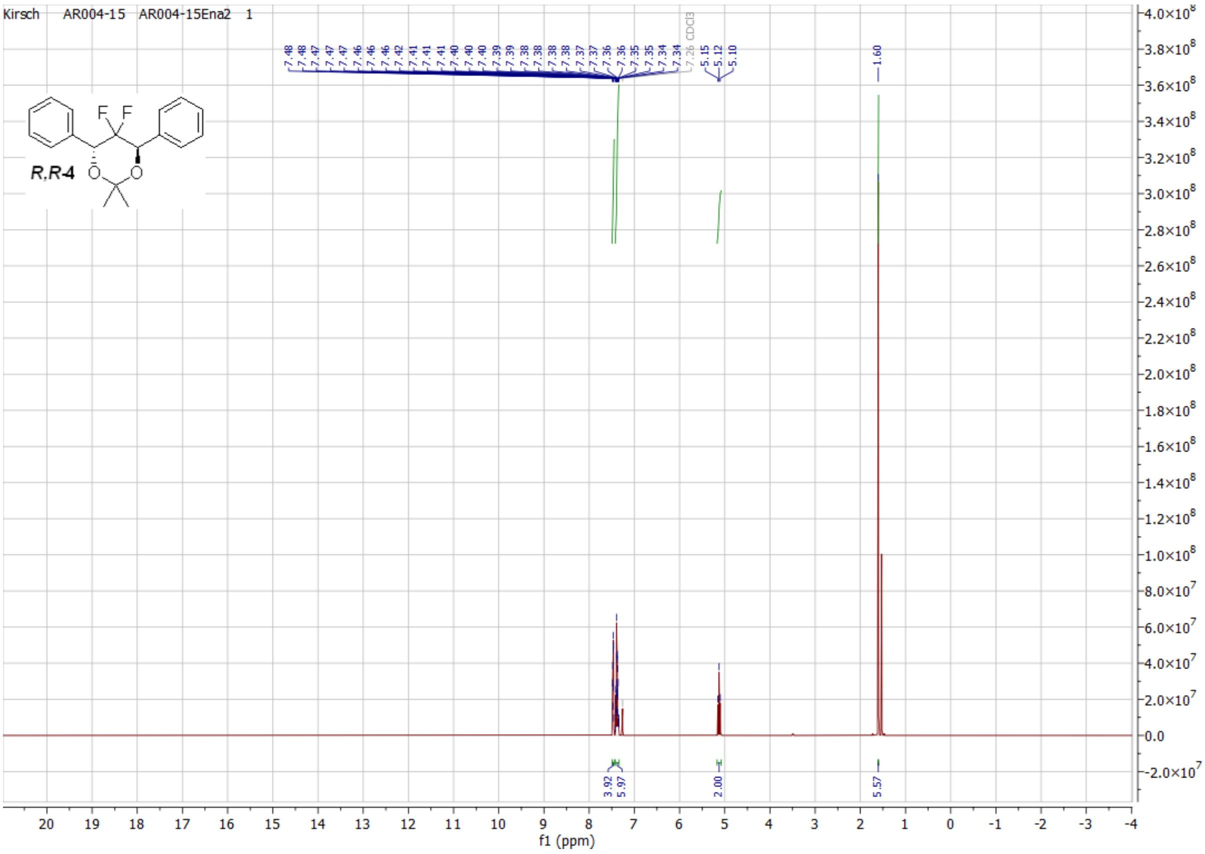

^{19}F NMR of (*R*,*R*)-**4**

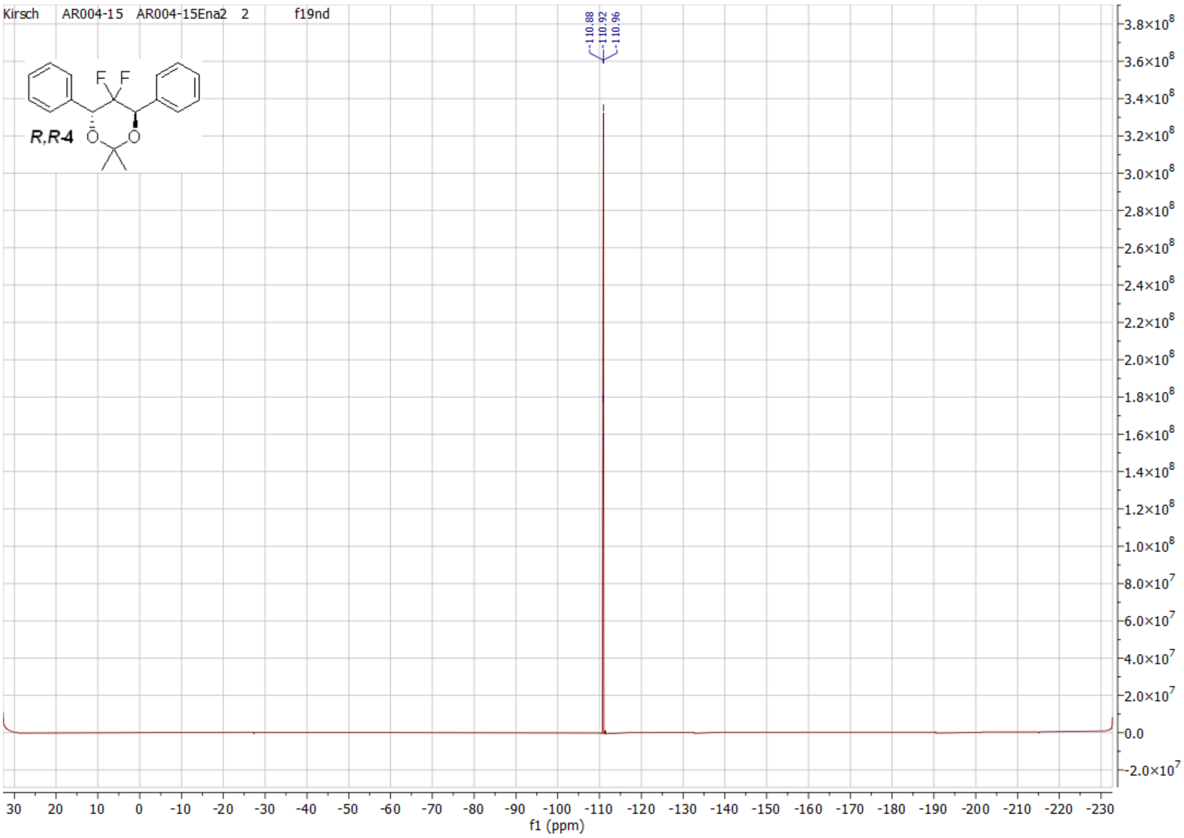

^{19}F NMR of (*R,R*)-**4**

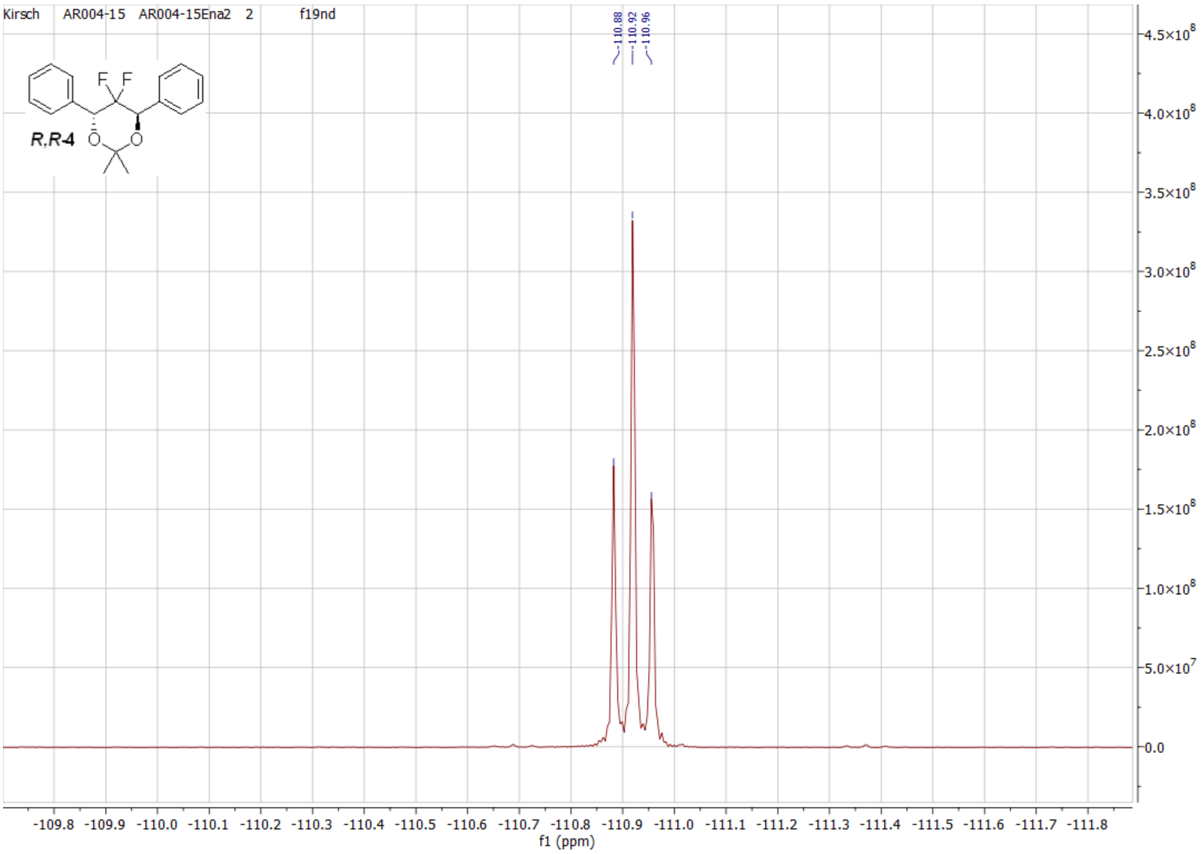

References

1. Xu, W.; Médebielle, M.; Bellance, M.-H.; Dolbier, Jr., W. R. *Adv. Synth. Catal*. **2010**, *352*, 2787-2790.

2. Kuroboshi, M.; Ishihara, T. *Bull. Chem. Soc. Jpn*. **1990**, *63*, 1185-1190.
